# C57BL/6J substrain differences in response to high-fat diet intervention

**DOI:** 10.1038/s41598-020-70765-w

**Published:** 2020-08-20

**Authors:** Majken Storm Siersbæk, Nicholas Ditzel, Eva Kildall Hejbøl, Stine Marie Præstholm, Lasse Kruse Markussen, Fabio Avolio, Lingzi Li, Lasse Lehtonen, Axel Kornerup Hansen, Henrik Daa Schrøder, Lukasz Krych, Susanne Mandrup, Louise Langhorn, Peter Bollen, Lars Grøntved

**Affiliations:** 1 Functional Genomics and Metabolism Research Unit, Department of Biochemistry and Molecular Biology, VILLUM Center for Bioanalytical Sciences, University of Southern Denmark, 5230 Odense M, Denmark; 2 Center for Functional Genomics and Tissue Plasticity (ATLAS), University of Southern Denmark, 5230 Odense M, Denmark; 3 Molecular Endocrinology Lab (KMEB), Department of Molecular Endocrinology, Odense University Hospital & University of Southern Denmark, 5000 Odense C, Denmark; 4 Department of Pathology, Odense University Hospital (OUH), 5000 Odense C, Denmark; 5 Department of Veterinary and Animal Sciences, Faculty of Health and Medical Sciences, University of Copenhagen, 1871 Frederiksberg C, Denmark; 6 Microbiology and Fermentation, Department of Food Science (UCPH FOOD), University of Copenhagen, 1958 Frederiksberg C, Denmark; 7 Biomedical Laboratory, University of Southern Denmark, 5000 Odense C, Denmark

**Keywords:** Experimental organisms, Metabolism, Endocrine system and metabolic diseases, Metabolic disorders, Endocrine system and metabolic diseases

## Abstract

C57BL/6J-related mouse strains are widely used animal models for diet-induced obesity (DIO). Multiple vendors breed C57BL/6J-related substrains which may introduce genetic drift and environmental confounders such as microbiome differences. To address potential vendor/substrain specific effects, we compared DIO of C57BL/6J-related substrains from three different vendors: C57BL/6J (Charles Rivers), C57BL/6JBomTac (Taconic Bioscience) and C57BL/6JRj (Janvier). After local acclimatization, DIO was induced by either a high-fat diet (HFD, 60% energy from fat) or western diet (WD, 42% energy from fat supplemented with fructose in the drinking water). All three groups on HFD gained a similar amount of total body weight, yet the relative amount of fat percentage and mass of inguinal- and epididymal white adipose tissue (iWAT and eWAT) was lower in C57BL/6JBomTac compared to the two other C57BL/6J-releated substrains. In contrast to HFD, the three groups on WD responded differently in terms of body weight gain, where C57BL/6J was particularly prone to WD. This was associated with a relative higher amount of eWAT, iWAT, and liver triglycerides. Although the HFD and WD had significant impact on the microbiota, we did not observe any major differences between the three groups of mice. Together, these data demonstrate significant differences in HFD- and WD-induced adiposity in C57BL/6J-related substrains, which should be considered in the design of animal DIO studies.

## Introduction

Experimental studies within fields of DIO and the following metabolic consequences most often involves in vivo mouse models. It is generally known that different commercially available inbred mouse strains do not respond similarly to HFD^1–5^. For example, whereas C57BL/6 usually gain weight on HFD, the 129SvEv strain is resistant to DIO^2,6^. Some of these differences may be explained by the genetic variance^7–9^. In addition, environmental factors such as housing facilities impacting the microbiota has been suggested to affect susceptibility to DIO^2,10,11^ (and reviewed in^12^). Importantly, multiple substrains of C57BL/6 are commercially available and many of these substrains are thus widely used in the field of obesity and diabetes research^13–15^. An extensive whole-genome comparison between the C57BL/6 substrains, C57BL/6J and C57BL/6N, identified several genetic variants, including several single nucleotide polymorphisms (SNPs)^7,8^ likely impacting physiology. Moreover, several studies have shown that C57BL/6J are more glucose intolerant than the C57BL/6N due to a functional deletion of exon 7–11 in the gene encoding nicotinamide nucleotide transhydrogenase (*Nnt*)^16–20^. NNT is an integral protein located in the inner mitochondrial membrane and it is a central source of mitochondrial NADPH important for reducing reactive oxygen species (ROS) necessary to maintain efficient ATP synthesis. In line with this, it has been reported that C57BL/6J have mitochondrial redox abnormalities and reduced insulin secretion^16,20,21^. However, it is highly debated whether the *Nnt* mutation affects DIO as some studies observed equal DIO response regardless of *Nnt* status^1,3^.

Interestingly, genetic differences, including the Nnt variant, have been reported in different C57BL/6J-related substrains^22^, emphasizing that C57BL/6J housed by different vendors are not necessarily genetically identical. For example, SNP analysis suggests that C57BL/6J and C57BL/6JArc separates from C57BL/6JBomTac, C57BL/6JRccHsd and C57BL/6JOlaHsd^22^. One of the SNPs (rs13477019) separating C57BL/6J-related substrains is shared between C57BL/6JBomTac and C57BL/6JRj^23^, implying that C57BL/6JRj separates from C57BL/6J but is similar to C57BL/6JBomTac, C57BL/6JRccHsd and C57BL/6JOlaHsd. However, the *Nnt* mutation is not shared between C57BL/6JBomTac and C57BL/6JRj, collectively suggesting that at least three genetically different C57BL/6J-related substrains exists; a) C57BL/6J and C57BL/6JArc, b) C57BL/6JBomTac, C57BL/6JRccHsd, and C57BL/6JOlaHsd and c) C57BL/6JRj. Although, previous studies have suggested an impaired DIO phenotype in C57BL/6JRj compared to C57BL/6NTac^4,24^, no direct comparison of DIO in mice derived from the original C57BL/6J strain, bred by different vendors, has been performed.

It is evident that gut microbiota (GM) influences the pathogenesis of obesity and insulin resistance and it has been suggested that an increased ratio of Firmicutes to Bacteroidetes (F/B) and decreased bacterial diversity are important markers of an obesogenic microbiome^25–30^. However, Ussar and colleagues reported similar low phylogenetic diversity in obesity- and diabetes-prone (C57BL/6J) and obesity- and diabetes-resistant (129SvEv/lmJ) mice and no correlation between F/B ratio^2,6,9,11^, suggesting that an obesogenic microbiome alone does not promote obesity. Hence, interplay between GM and host genetics, local housing environment, early life exposure, and diet composition has also been shown to highly influence GM diversity and metabolic profile^2,6,9–11,31^. This suggests, that composition of the GM is highly dynamic, is affected by numerous factors, and either is a cause or a consequence of metabolism. Thus, C57BL/6J-related strains bred by different vendors may harbor diverse microbiota, which together with the genetical variability may impact DIO.

To evaluate DIO in different C57BL/6J-related substrains we focused on three seeming genetically distinct substrains donated by three different vendors: C57BL/6JBomTac (from Taconic Bioscience), C57BL/6J (distributed by SCANBUR and bred on Charles Rivers license) and C57BL/6JRj (from Janvier). DIO was induced by two different diets for 10 weeks: HFD (60% energy from fat) or WD (42% energy from fat supplemented with fructose in the drinking water). Importantly, mice in this study were not bred in the local animal facility but were subjected to diet intervention after two weeks of acclimatization. Mice were thoroughly evaluated for body weight gain during diet intervention, glucose- and insulin tolerance tests (GTT and ITT), dual-energy X-ray absorptiometry (DEXA) scans, and finally liver, pancreas, spleen, kidney, epididymal (visceral) or inguinal (subcutaneous) white adipose tissue (eWAT and iWAT, respectively) were dissected and weighted, histology performed and GM from feces and cecum samples were sequenced. We found major vendor-specific differences in regard to body weight gain, depending on the type of high-fat diet, and differences in the storage of excess fat and composition of GM. Overall, C57BL/6J displayed significant increase in body weight on WD, were less hyperinsulinaemic on HFD and stored more of the excess energy from both high-fat diets in visceral and subcutaneous adipose tissue and liver compared to C57BL/6JRj and C57BL/6JBomTac. C57BL/6JRj were less prone to DIO on WD and displayed a healthier metabolic profile (lower hyperinsulinemia) compared to C57BL/6JBomTac and C57BL/6J on WD. Although we observed a strong impact of DIO on the microbiota only modest differences were observed between C57BL/6J, C57BL/6JRj and C57BL/6JBomTac, suggesting that the microbiome is likely not involved in the observed physiological difference.

## Results

### Experimental setup

**Figure 1:**
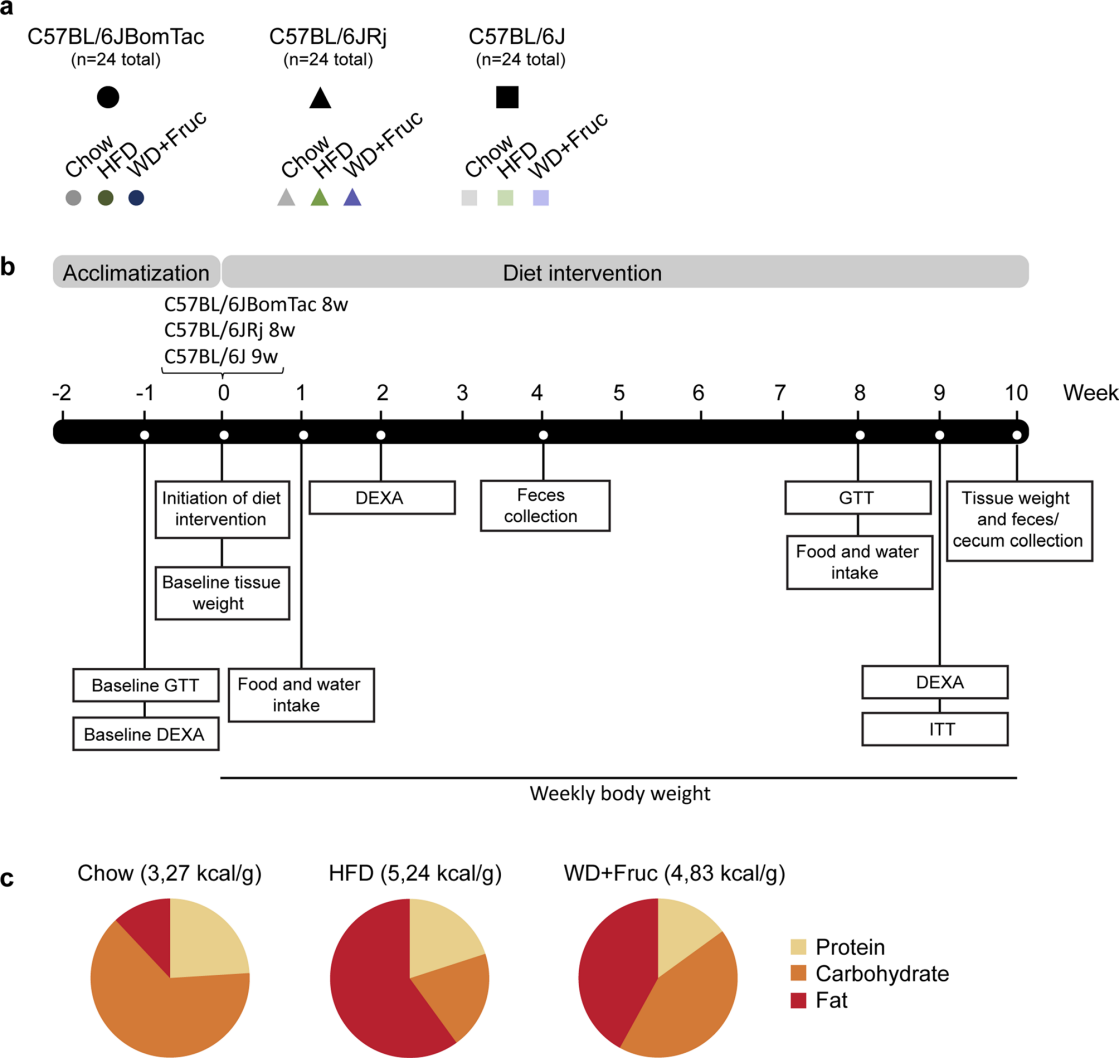
Experimental setup. (**a**) C57BL/6JBomTac (filled circle Taconic Bioscience), C57BL/6JRj (filled triangle Janvier), and C57BL/6J (filled rectangle Charles Rivers), arrived at six to seven weeks of age. (**b**) After two weeks of acclimatization, a baseline group (n = 6) from each strain was subjected to DEXA scanning and GTT before being sacrificed. Subsequently, 10 weeks diet intervention was initiated on the remaining mice; one group of each strain continued on control chow diet (n = 6), one group changed to a high-fat-diet (HFD) (n = 6) and one group changed to a Western diet (WD) (n = 6) with fructose in the drinking water. At week one and eight average food and water intake was estimated. At week eight GTT was performed. At week two and nine DEXA scanning was performed, and at week nine ITT was performed. Body weight was measured weekly. After 10 weeks of diet intervention mice were sacrificed. (**c**) Macronutrient content of the chow, HFD and WD diets. During diet intervention some mice succumbed resulting in n < 6 for some treatment groups at the end of the diet intervention.

C57BL/6J-related substrains were sponsored by three different vendors and acclimatized at the local animal facility before onset of HFD and WD intervention (Fig. 1a). After two weeks of acclimatization at the local animal facility, we randomized the mice and performed baseline intraperitoneal glucose tolerance test (GTT) and DEXA scan, and measured the weight of liver, iWAT, eWAT, spleen, and pancreas (Fig. 1b). At eight to nine weeks of age mice from the three different vendors were challenged either with chow diet, HFD (60% energy from fat) or WD (42% energy from fat and fructose in the drinking water) (Fig. 1a,c) for 10 weeks, which has been shown to cause significant physiological and metabolic changes^2,10^ (see Suppl. Table 1 for macro- and micronutrient content of diets). After one and eight weeks of diet intervention average food and water intake were estimated. Intraperitoneal GTT was performed after eight weeks, DEXA scan was carried out after two and nine weeks, intraperitoneal insulin tolerance test (ITT) after nine weeks, feces samples were collected after 4 and 10 weeks and cecum samples after 10 weeks (summarized in Fig. 1b). Body weight was measured every week of diet intervention. After 10 weeks of diet intervention we isolated liver, iWAT, eWAT, spleen, and pancreas, performed liver, iWAT, and eWAT histology and analyzed the GM.

### C57BL/6JRj have lower body and liver weight and lower fasting serum insulin compared to C57BL/6JBomTac and C57BL/6J at baseline sacrifice

**Figure 2:**
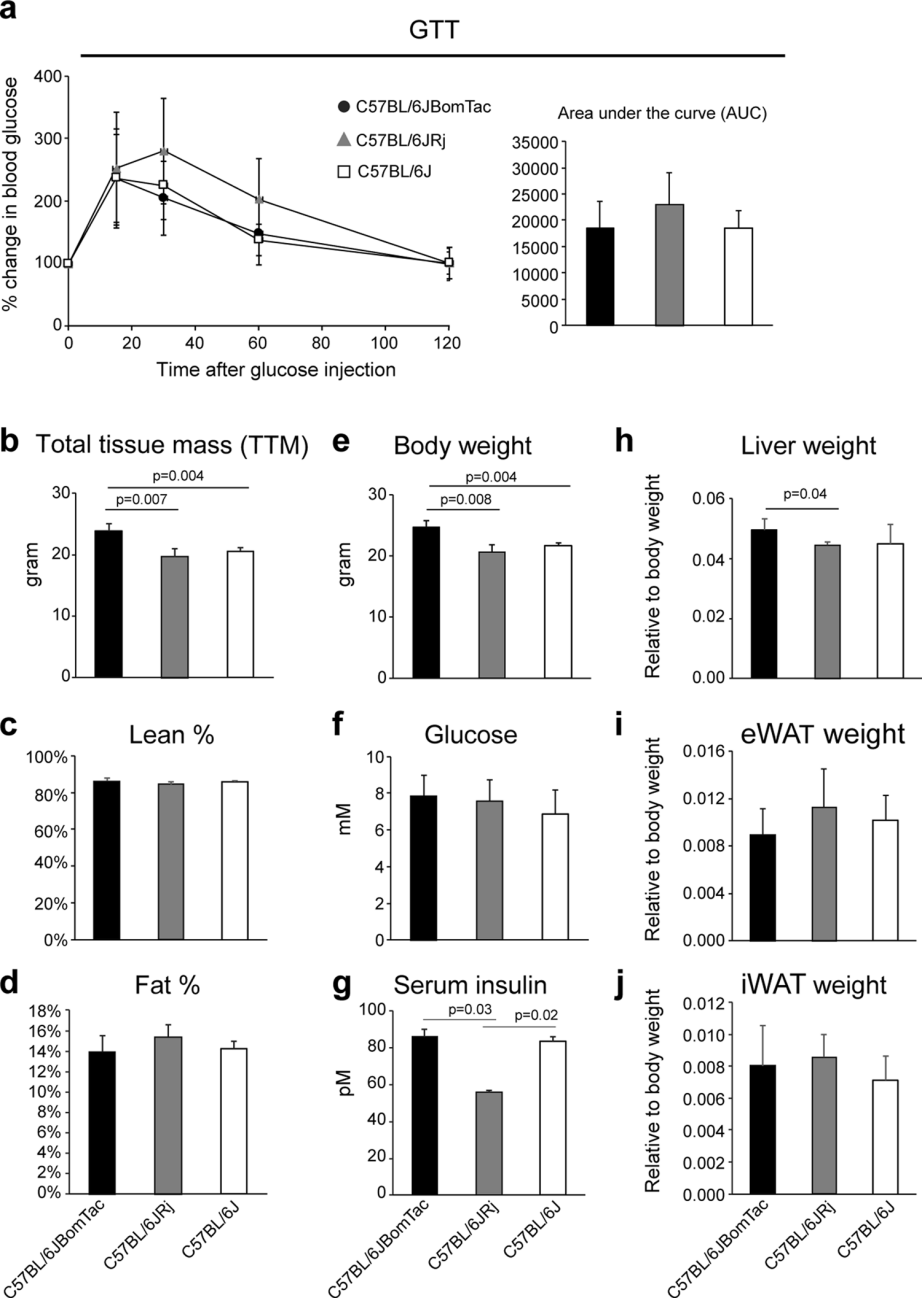
Baseline sacrifice. (**a**) GTT in C57BL/6JBomTac (filled circle), C57BL/6JRj (filled triangle), and C57BL/6J (filled rectangle) before baseline sacrifice (left) and area under the curve (AUC) (right), n = 6 for each group. (**b**–**d**) DEXA scan of baseline groups (n = 6) measuring total tissue mass (TTM), lean % and fat %. (**e**–**j**) Sacrifice of the baseline groups (n = 6) was initiated after 4-7 h of fasting (see experimental procedures) and (**e**) body weight, (**f**) fasting blood glucose, (**c**) fasting serum insulin, and weight of (**h**) liver, (**i**) eWAT, and (**j**) iWAT was measured. Data is presented as mean ± SD. Statistical significance is calculated using one-way ANOVA with an n = 6 and p-values are shown in the figure.

To assess potential differences before diet intervention, we performed GTT and DEXA scan after acclimatization at the local animal facility. As shown in Fig. 2a there were no significant differences between the three groups of mice regarding handling of a high glucose load (GTT) before initiation of diet intervention. DEXA scan revealed that C57BL/6JRj and C57BL/6J had significantly lower total tissue mass (TTM) compared to C57BL/6JBomTac (Fig. 2b). Yet, no significant differences were observed in lean and fat % (Fig. 2c,d) or bone parameters (Suppl. Fig. 1a). Moreover, tibia length relative to body weight was lower for C57BL/6JBomTac compared to C57BL/6J and C57BL/6JRj, suggesting that C57BL/6JBomTac mice were not heavier due to larger body length (Suppl. Fig. 1b). However, tibia length may not be the optimal estimate of body length and we cannot exclude that the C57BL/6JBomTac mice were larger in size at the beginning of the study. In accordance with TTM, the body weight of C57BL/6JRj and C57BL/6J were significantly lower than C57BL/6JBomTac (Fig. 2e), which was expected based on publicly available growth curves from the vendors. We did not observe a significant difference in fasting glucose levels between the three groups of mice, however, C57BL/6JRj had significantly lower fasting serum insulin levels compared to C57BL/6JBomTac and C57BL/6J (Fig. 2f,g). In addition, C57BL/6JRj had significantly lower liver weight relative to body weight compared to C57BL/6JBomTac (Fig. 2h) but there were no significant differences in the weight of eWAT and iWAT depots between the three C57BL/6J-related substrains at baseline (Fig. 2i,j). Furthermore, the weight of the spleen was not significantly different between the three groups of mice, but C57BL/6J mice had significantly higher pancreas weight compared to C57BL/6JRj (Suppl. Fig. 1c). Overall, C57BL/6JRj displayed lower body and liver weight and lower fasting serum insulin levels compared to C57BL/6JBomTac and/or C57BL/6J at baseline sacrifice.

### Significant differences in body weight gain and body composition during diet intervention between C57BL/6J-related substrains

**Figure 3:**
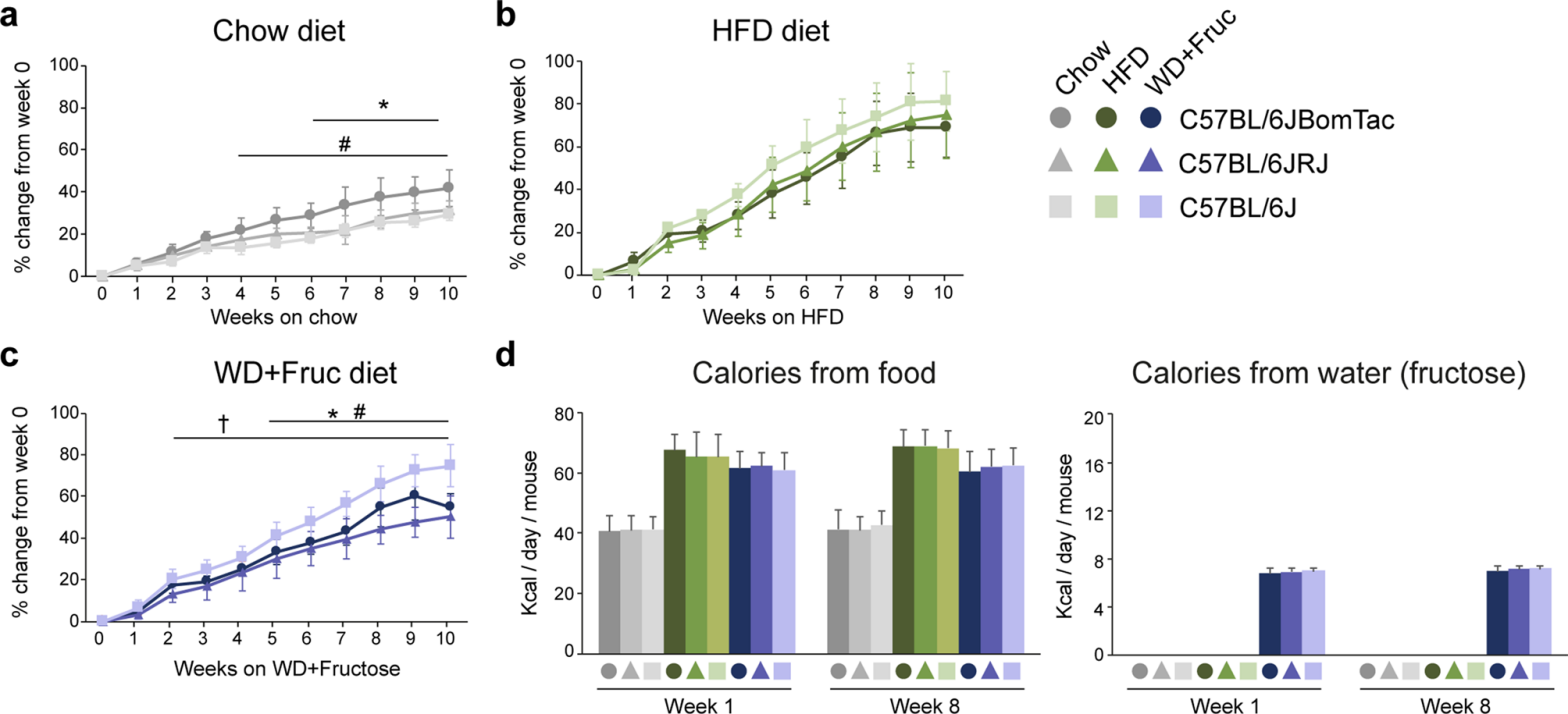
Comparison of body weight in C57BL/6J-related substrains on chow, HFD, and WD. Body weight gain, shown as percentage change from week 0, during 10 weeks of diet intervention. (**a**) comparison of chow groups, (**b**) HFD groups, and (**c**) WD groups with fructose in drinking water. (**d**) Summed calorie intake from food and drinking water at week one and week eight of diet intervention. Data is presented as mean ± SD. Statistical significance is calculated using two-way ANOVA with n = 4–6. Statistical significance for a-c) is indicated comparing the different strains (a) C57BL/6JRj (filled triangle) vs C57BL/6JBomTac (filled circle), (b) C57BL/6J (filled rectangle) vs. C57BL/6JBomTac (filled circle) or (c) C57BL/6JRj (filled triangle) vs. C57BL/6J (filled rectangle). P-values < 0.05 are indicated for (*) C57BL/6JRj (filled triangle) vs C57BL/6JBomTac (filled circle), (^#^) C57BL/6J (filled rectangle) vs. C57BL/6JBomTac (filled circle) or (^†^) C57BL/6JRj (filled triangle) vs. C57BL/6J (filled rectangle).

To investigate temporal macroscopic effects of diet intervention of the three groups of mice we measured body weight on a weekly basis. Since the initial weight of C57BL/6JBomTac was significantly different from the C57BL/6JRj and C57BL/6J (Fig. 2b,e) we normalized the body weight of the mice for all three diet intervention groups to their individual body weight at the start of diet intervention (Fig. 3a–c). Unnormalized body weight measurements are shown in Suppl. Fig. 2a–c. Interestingly, after four and six weeks on chow diet C57BL/6JBomTac gained significantly more weight than C57BL/6J and C57BL/6JRj, respectively (Fig. 3a). The same tendency was observed in absolute body weight gain (Suppl. Fig. 2a). Interestingly, there were no significant differences in weight gain between mice on HFD (Fig. 3b and Suppl. Fig. 2b), however, in contrast, C57BL/6J gained significantly more body weight after two and five weeks on WD compared to C57BL/6JRj and C57BL/6JBomTac, respectively (Fig. 3c). In addition, C57BL/6JBomTac also gained significantly more weight after five weeks on WD compared to C57BL/6JRj (Fig. 3c), suggesting that the C57BL/6JRj is less susceptible to WD induced obesity compared to C57BL/6JBomTac and C57BL/6J. Interestingly, C57BL/6J gained significant more weight on HFD and WD compared to chow already after two weeks of diet intervention, whereas significant differences in body weight could not be observed until five and six weeks after diet intervention for C57BL/6JBomTac and C57BL/6JRj, respectively (Suppl. Fig. 2d–f). This demonstrates that the DIO induced weight gain is accelerated in C57BL/6J compared to C57BL/6JBomTac and C57BL/6JRj.

To investigate whether differences in body weight gain was due to differences in calorie intake, we estimated food and water intake in the early (week one) and late (week eight) phase of the diet intervention. As expected, mice on HFD and WD had increased caloric intake, however we did not observe any significant differences between the substrains (Fig. 3d and Suppl. Fig. 2g). Thus, the relatively less pronounced weight gain observed for C57BL/6JRj on WD cannot be explained by less caloric intake. Comparing the chow, HFD, and WD in each of the three C57BL/6J-related substrains underlined this observation as C57BL/6JBomTac and C57BL/6J gained similar amount of body weight on HFD and WD with equal calorie intake whereas C57BL/6JRj gained significantly less body weight on WD compared to HFD despite similar calorie intake on the two diets (Suppl. Fig. 2d–g).

To investigate if differences in body weight could be explained by changes in body composition, we performed DEXA scans at nine weeks of diet intervention. In agreement with the general increase in body weight upon HFD and WD, we observe increased total tissue mass for all substrains as a result of diet intervention (Suppl. Fig. 3a–c). On HFD all three groups of mice showed increased percentage of fat compared to chow, in agreement with the similar increased weight gain as a result of HFD (Suppl. Fig. 3a–c). In contrast, whereas WD resulted in increased fat percentage for C57BL/6JBomTac and C57BL/6J, no significant change in fat percentage was observed for C57BL/6JRj on WD compared to chow (Suppl. Fig. 3b). This agrees with the reduced susceptibility to DIO by WD in C57BL/6JRj compared to the mice from two other vendors. Only minor differences in bone parameters were observed between the three groups of mice. In general bone mineral density (BMD), bone mineral content (BMC) and bone area (BA) was higher in C57BL/6JBomTac after nine weeks on chow (Suppl. Fig. 3d–f) in agreement with higher body weight (Fig. 3a and Suppl. Fig. 2a). Interestingly, tibia length relative to body weight was higher for C57BL/6JRj and C57BL/6J mice on chow compared to C57BL/6JBomTac which resembled baseline values (Suppl. Fig. 1b). However, on WD C57BL/6JRj had a longer tibia length relative to body weight compared to C57BL/6JBomTac and C57BL/6J (Suppl. Fig. 3g).

### The C57BL/6J-related substrains display no differences in insulin- and glucose tolerance during 10 weeks of diet intervention

**Figure 4:**
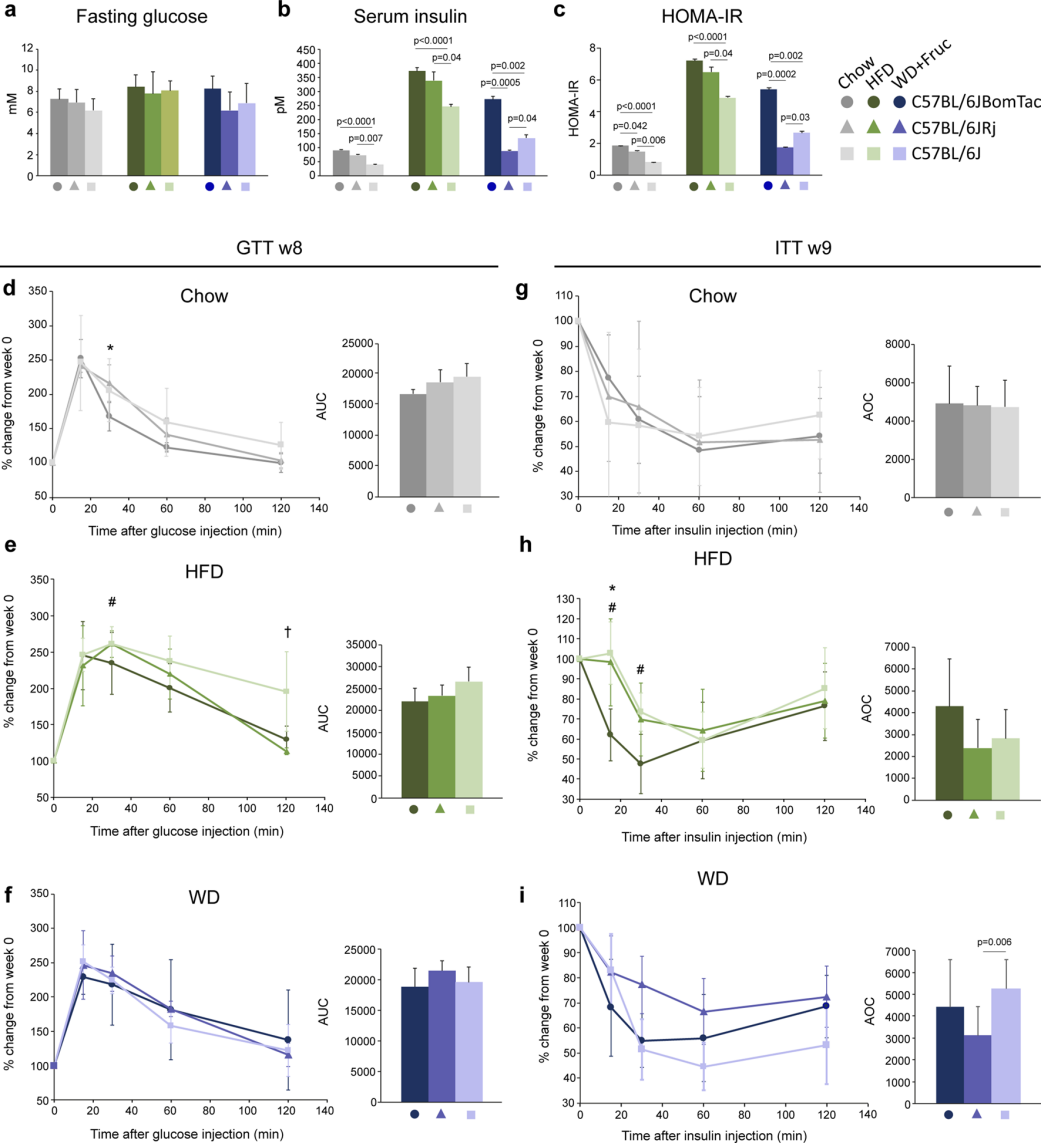
Glucose and insulin levels and tolerance test after eight to nine weeks of high-fat diet feeding. (**a**) Fasting blood glucose, (**b**) serum insulin, and **c**) HOMA-IR in C57BL/6JBomTac (filled circle), C57BL/6JRj (filled triangle), and C57BL/6J (filled rectangle) on chow, HFD, and WD + Fructose after 10 weeks of diet intervention. (**d**–**i**) Glucose and insulin tolerance test, shown in percentage change from week 0, in C57BL/6JBomTac (filled circle), C57BL/6JRj (filled triangle), and C57BL/6J (filled rectangle) on (**d**, **g**) chow, (**e**, **h**) HFD, and (**f**, **i**) WD + Fructose (left) and AUC/AOC (right) after eight or nine weeks of diet intervention. Data is presented as mean ± SD. Statistical significance is calculated using two-way ANOVA with n = 4–6. P-values for a-c) are shown in the figure. Significance (**d**–**i**) is indicated comparing the different strains p < 0,05 (*) C57BL/6JRj (filled triangle) vs C57BL/6JBomTac (filled circle), (^#^) C57BL/6J (filled rectangle) vs. C57BL/6JBomTac (filled circle) or (^†^) p < C57BL/6JRj (filled triangle) vs. C57BL/6J (filled rectangle).

In order to examine the metabolic profile of the three groups of mice we measured fasting blood glucose and serum insulin levels and calculated HOMA-IR. We found no significant differences in fasting blood glucose levels after 10 weeks of diet intervention (Fig. 4a), however, C57BL/6J displayed significantly lower fasting insulin and HOMA-IR on both chow, HFD, and WD compared to C57BL/6JBomTac (Fig. 4b,c). Interestingly, in line with the observation that C57BL/6JRj on WD gained less weight compared to the two other groups of mice, C57BL/6JRj displayed lower fasting serum insulin levels and HOMA-IR compared to both C57BL/6JBomTac and C57BL/6J (Fig. 3c, Suppl. Figs. 3a–c, 4b,c). This suggests that despite similar, although accelerated, weight gain on HFD, C57BL/6J may be more insulin sensitive compared to C57BL/6JBomTac and C57BL/6JRj and despite higher weight gain on WD, C57BL/6J may be less insulin resistant than C57BL/6JBomTac.

To investigate if the altered fasting serum insulin affected glucose and insulin tolerance, we performed intraperitoneal GTT at week eight and ITT at week nine of diet intervention. Due to variations in individual mouse blood glucose values between diet groups data was normalized to week 0 values (% change from week 0, total glucose levels are shown in Suppl. Fig. 4). As expected, both C57BL/6JBomTac and C57BL/6J on HFD displayed slower glucose clearance (area under the curve (AUC)) compared to chow groups (Suppl. Fig. 5a,c). Same tendency applied to glucose clearance in C57BL/6JRj but did not reach statistical significance of p < 0.05 (Suppl. Fig. 5). In contrast, mice fed WD did not obtain significant differences in glucose clearance compared to chow diet in any of the three groups of mice (Suppl. Fig. 5a–c). Furthermore, there was a tendency towards decreased insulin sensitivity at a few time points during ITT in C57BL/6J fed HFD compared to mice fed chow diet, but when calculating area over the curve (AOC) it only reached statistical significance between chow and HFD groups in C57BL/6JRj and between HFD and WD groups in C57BL/6J mice (Suppl. Fig. 5d–f). No significant differences in insulin tolerance (AOC) were observed in C57BL/6JBomTac (Suppl. Fig. 5d).

When comparing the three groups of mice on chow, HFD, and WD, respectively, there was no significant differences in glucose tolerance (glucose AUC) (Fig. 4d–i), however, C57BL/6J on HFD tended to have a slower glucose clearance than C57BL/6JBomTac and C57BL/6JRj (Fig. 4e). Likewise, there was no significant insulin tolerance (insulin AOC) differences between the mice on chow and HFD, respectively, however, C57BL/6J on WD had significantly higher glucose clearance after insulin injection compared to C57BL/6JRj (Fig. 4i). Overall this demonstrates, that despite vendor-specific differences in body weight and body composition, there were only minor differences in glucose and insulin tolerance after eight-nine weeks of diet intervention. Most likely, potential differences would need longer diet intervention time to develop and/or large group size to show significant differences. Thus, despite little differences in GTT and ITT, the fasting insulin levels of C57BL/6JRj on WD suggest an absence of hyperinsulinemia compared to C57BL/6JBomTac and C57BL/6J which complies with C57BL/6JRj being less susceptible to WD induced obesity.

### Major C57BL/6J vendor-specific differences in organ weight and hepatic triglyceride accumulation after 10 weeks of diet intervention

**Figure 5:**
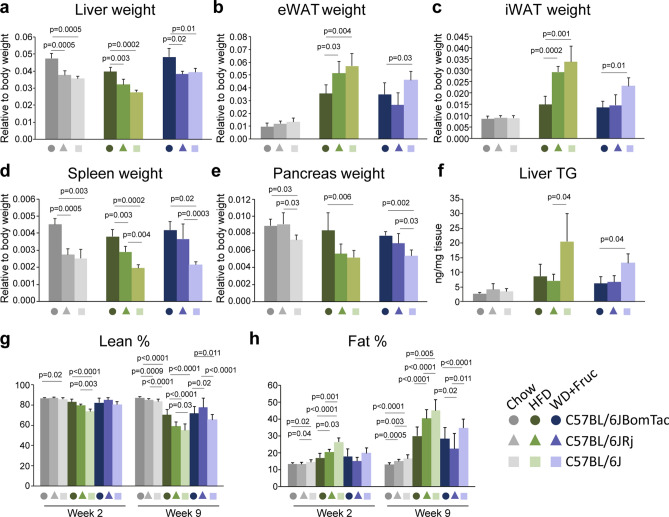
Weight of liver, eWAT, iWAT, spleen, and pancreas, liver TG quantification and body composition measurements after 10 weeks of high-fat diet intervention. Weight of (**a**) liver, (**b**) eWAT, (**c**) iWAT, (**d**) spleen, (**e**) pancreas, and (**f**) liver TG levels after 10 weeks of diet intervention. Body composition was evaluated using DEXA scans (**g**) lean % and (**h**) fat % after one and nine weeks of diet intervention. Data is presented as mean ± SD. Statistical significance is calculated using one-way ANOVA with n = 4–6. P-values are shown in the figure.

To investigate if the observed differences in body weight gain of the three groups of mice are reflected in the weight of various tissues, mice were sacrificed after 10 weeks of diet intervention and various tissues were dissected. Interestingly, we found significant differences in the weight of five major tissues; the liver, eWAT and iWAT depots, spleen, and pancreas both when plotted as relative to body weight (Fig. 5a–e) and as absolute values (Suppl. Fig. 6a–e). C57BL/6JBomTac had significantly higher liver weight compared to C57BL/6JRj and C57BL/6J, both on chow, HFD, and WD (Fig. 5a and Suppl. Fig. 6a). This may be related to differential glycogen content (Suppl. Fig. 6f). Interestingly, however, C57BL/6J store significantly more fat in the liver than C57BL/6JBomTac and C57BL/6JRj both on HFD and WD quantified by liver triglycerides (TG) (Fig. 5f) and visualized in Oil-Red-O stainings of liver sections (Suppl. Fig. 6g).

In contrast to high liver weight, C57BL/6JBomTac had lower weight of eWAT and iWAT depots on HFD compared to the C57BL/6JRj and C57BL/6J (Fig. 5b,c). This agrees with a relative lower fat % and higher lean % in C57BL/6JBomTac measured by DEXA (Fig. 5g,h). This difference was less pronounced for mice on WD, although a tendency for higher fat mass and fat % could be observed in C57BL/6J compared to the two other C57BL/6J-related strains (Fig. 5b,c,h). Measurement of adipocyte tissue size showed no significant differences in iWAT and eWAT (Suppl. Fig. 7), suggesting that differences are a result of change in the number adipocytes.

Like liver, spleen and pancreas weight were significantly higher in C57BL/6JBomTac on chow, HFD, and WD compared to either one or both of the two other groups of mice (Fig. 5d,e and Suppl. Fig. 6d,e). Moreover, in contrast to WAT weight, C57BL/6J had a lower weight of the spleen on HFD and WD, and lower weight of the pancreas on chow and WD compared to mice from the two other vendors (Fig. 5d,e).

Furthermore, there were also vendor-specific differences in response to HFD and WD, summarized in Suppl. Fig. 8. To highlight a few, C57BL/6JBomTac and C57BL/6J showed a significant increase in liver TG on HFD and WD compared to chow, whereas no significant differences were detected in C57BL/6JRj mice (Suppl. Fig. 8a–c). Moreover, all three groups of mice had lower weight of iWAT on WD compared to HFD whereas this was only the case for C57BL/6JRj in the eWAT (Suppl. Fig. 8a–c).

Collectively, these data reveal major differences between C57BL/6J-related substrains from different vendors regarding weight of liver, eWAT, iWAT, spleen, and pancreas. C57BL/6JRj and C57BL/6J show a more pronounced expansion of the adipocyte depots in response to HFD compared to C57BL/6JBomTac. Interestingly, this tendency was not replicated for C57BL/6JRj on WD. Here C57BL/6JBomTac and C57BL/6JRj had lower adipose tissue mass compared to C57BL/6J. Thus, a general tendency was that C57BL/6J consistently had a higher adipose tissue mass as a result of DIO. This tendency was also observed for TG storage in the liver. This aligns well with the general more pronounced weight gain observed for C57BL/6J on WD. In contrast, we did not observe any clear diet-induced tissue weight and liver TG difference between C57BL/6JRj and the two other groups of mice, suggesting that the mass of the different measured tissue does not explain the less pronounced weight gain for C57BL/6JRj.

### Microbiome composition in C57BL/6J-related substrain as a result of DIO

**Figure 6:**
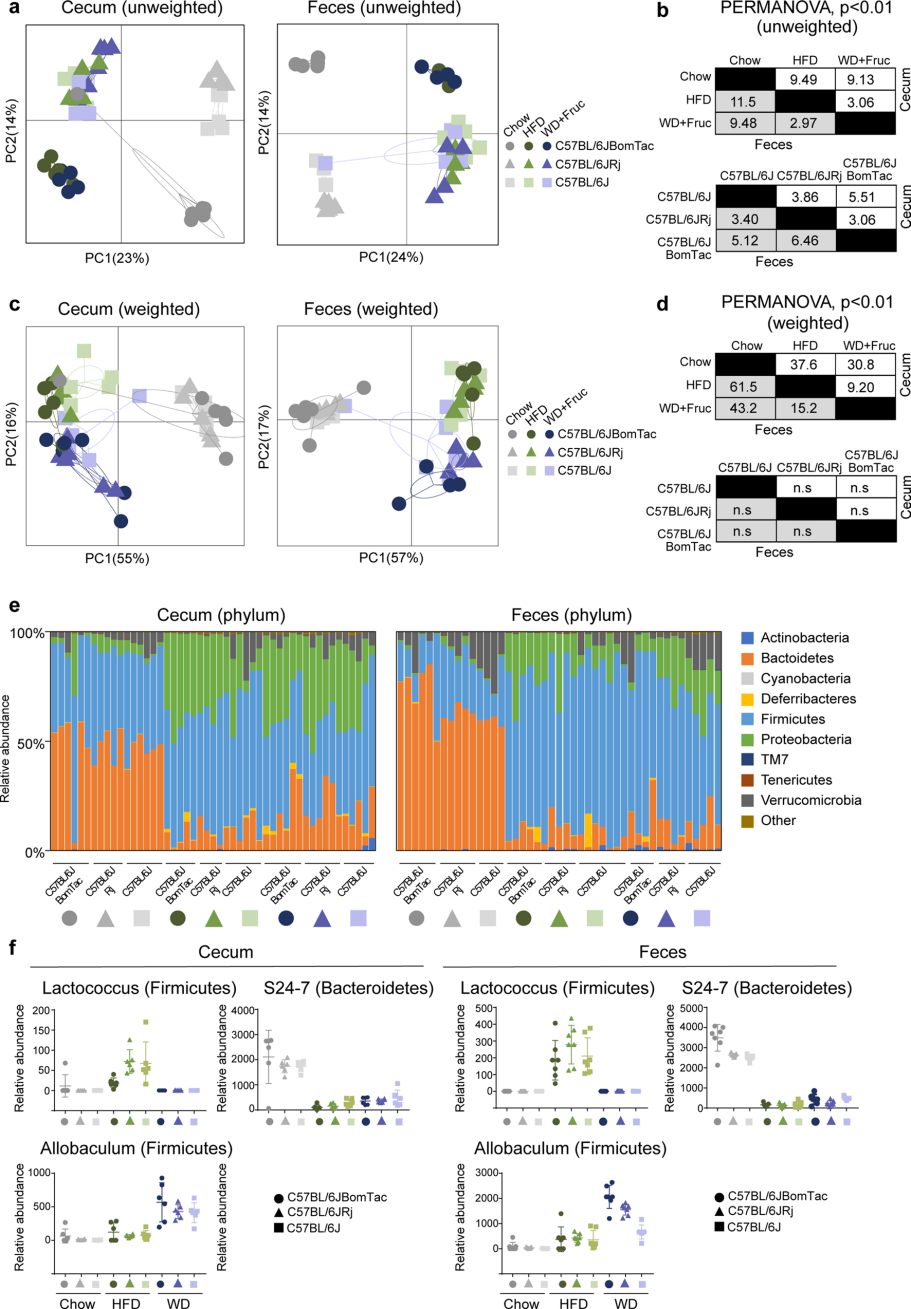
Gut microbiota C57BL/6J-related substrains. PCoA of (**a**) unweighted UniFrac distances for feces (n = 67) and cecum (n = 65) samples from C57BL/6JBomTac (filled circle), C57BL/6JRj (filled triangle), and C57BL/6J (filled rectangle) and (**c**) weighted UniFrac distances. (**b**, **d**) Corresponding PERMANOVA results for unweighted and weighted UniFrac distances. (**e**) Relative abundance of bacteria phyla within cecum and feces samples from C57BL/6JBomTac (filled circle), C57BL/6JRj (filled triangle), and C57BL/6J (filled rectangle). (**f**) Selected bacteria taxa significantly different (p < 0.05 in ANCOM test) between diets (Lactococcus, Prevotella S24-7, and Allobaculum).

As metabolism is tightly coupled to the composition of the microbiome^11,12^, especially during diet intervention, we identified the gut microbial composition from feces and cecum samples from mice after four and 10 weeks of diet intervention. 16S rRNA gene amplicon sequencing identified a total of 77 different taxonomic groups representing six different phyla. Principal Coordinate Analysis (PCoA) based on UniFrac distance matrices from ceca and feces showed clear differences in microbiota composition both qualitative (unweighted UniFrac distances) and quantitative (weighted UniFrac distances) in response to DIO induced by HFD and WD (Fig. 6a–d). Vendor and diet differences were confirmed by PERMANOVA (Fig. 6b,d). Analysis based on unweighted UniFrac distance matrices of ceca and fecal microbiota showed clear diet and vendor differences, where C57BL/6JBomTac clearly separated from C57BL/6JRj and C57BL/6J and mice on chow generally separate from mice on HFD and WD (Fig. 6a,b). However, although weighted UniFrac distances, describing abundance of microbiota, revealed clear differences between lean and obese, no significant differences according to vendor was observed (Fig. 6c,d). This implies that diet had the strongest effect on microbiota composition, yet vendor effects were still observed and mostly allocated in taxa presenting lower abundance.

Comparison of bacterial relative abundance with Analysis of Composition of Microbes (ANCOM) tests on the phylum level, demonstrated diet-related differences in Proteobacteria, Firmicutes and Bacteroidetes, where Bacteroidetes were less abundant in HFD and/or WD, and Proteobacteria and Firmicutes were more abundant compared to chow consistent with previous reports^2,11^ (Fig. 6e and suppl. Fig. 9a–f). Although no significant phylum level difference (using ANCOM tests) were observed between the three susbtrains, we found some differences by ANOVA tests (Suppl. Fig. 9). For example, Proteobacteria was less abundant in cecum and feces from C57BL/6J compared to C57BL/6JBomTac on HFD. The species level ANCOM tests showed differences in the relative abundance of Bifidobacterium (Actinobacteria), Prevotella (Bacteroidetes), Allobaculum (Firmicutes), Lactococcus (Firmicutes) and Sutterella (Proteobacteria) in response to HFD and/or WD. For example, among the members of Firmicutes, the abundance of Lactococcus was reduced in WD group in favor of Allobaculum that was elevated in WD compared to chow and HFD (Fig. 6f). Also, the reduced level of Bacteroidetes upon HFD and WD (Suppl. Fig. 9e–f) could be assigned to reduced level of S24-7 (Fig. 6f). Collectively, this suggests that HFD and WD induced obesity lead some unique features of microbiome composition, however no clear substrain specific effects could be observed.

## Discussion

C57BL/6J-related inbred mice are widely used animal models to study DIO, where numerous commercial animal facilities provide C57BL/6J-related substrains to the research community. Thus, it is important to discriminate between C57BL/6J-related substrains supplied by different vendors. For example, as a result of unavoidable environmental differences between the commercial animal facilities it is possible that C57BL/6J-related substrains purchased from different vendors may respond differently to diet intervention in a local animal facility. Moreover, the reported genetic differences between some C57BL/6J-related substrains^8,16–19^ could contribute to the response to HFD and WD. Such putative differences will be important to consider in the design of animal experiments. In this study, we investigated the differences in DIO between genetically different C57BL/6J-related substrains from three different vendors and found numerous significant differences. One important observation was that C57BL/6JBomTac (*Nnt* wildtype) gained significantly more body weight on chow diet compared to C57BL/6JRj and C57BL/6J (both *Nnt* mutants). This observation is in line with previous publications comparing a *Nnt*-loss of function mice with a *Nnt* wildtype, suggesting that *Nnt*-loss of function might affect metabolism during normal chow feeding as opposed to/in addition to HFD^1,3^. Interestingly however, all three groups of mice gained a similar amount of body weight on HFD, supporting observations that *Nnt*-loss of function is clearly not the sole driver of DIO sensitivity^1,3^. Thus, on HFD C57BL/6JRj mice were equally susceptible to DIO compared to C57BL/6JBomTac and C57BL/6J. This disagrees with previous reports showing a protection against DIO in C57BL/6JRj when compared with C57BL/6NTac^4^.

Moreover, comparing the three groups of mice showed that C57BL/6J gained significantly more weight on WD relative to chow compared to C57BL/6JRj and C57BL/6JBomTac. This has, to our knowledge, not previously been shown and suggests that Nnt mutations is not a contributing factor to DIO in general. Interestingly, C57BL/6JRj on WD gained less weight and had lower fat percentage compared to HFD, despite same calorie intake. Similar to the C57BL/6JRj, C57BL/6J had lower fat percentage on WD compared to HFD, although C57BL/6J on WD gained similar body weight as HFD group. Interestingly, all three groups of mice on WD had lower adipose tissue mass and higher liver weight compared to HFD groups, which is consistent with previous reports showing that WD with time leads to more pronounced non-alcoholic fatty acid liver disease with e.g. high amount of fat accumulation in the liver^25^ (reviewed in^26^). Thus, if the aim of a study is to induce pronounced liver steatosis it may be most optimal to choose C57BL/6J. However, if the aim is to maximize adipose tissue mass C57BL/6JRj or C57BL/6J on HFD seem the best choice. Importantly, pronounced increased adipose mass is not observed in C57BL/6JBomTac and C57BL/6JRj on a WD and C57BL/6JRj is less susceptible to DIO on WD, which should be considered when choosing these mice as a WD obesity model.

In contrast to differences between the three C57BL/6J-related substrains observed in weight gain and tissue mass, we did not observe any major differences in GTT and ITT. However, due to the relatively short time of diet intervention and relative low number of animals in certain treatment groups we cannot exclude a possible difference in insulin sensitivity. We did, however, observe a difference in fasting insulin levels, where C57BL/6J showed less pronounced hyperinsulinemia as a result of HFD compared to C57BL/6JBomTac and C57BL/6JRj. Similarly, on WD C57BL/6JBomTac showed more pronounced fasting insulin levels compared to C57BL/6JRj and C57BL/6J, suggesting that C57BL/6JBomTac may be more susceptible to DIO induced hyperinsulinemia compared to mice from the other two vendors, however since the number of animals is relatively low this needs to be verified by additional studies. Thus, the degree of whole-body insulin sensitivity is not obviously different between the three groups of mice.

It is well known that obesity and/or T2D is reflected in gut microbiota composition^2,11,29,30,32^. For example, quantification of microbiota at the phylum level has shown increased (F/B) ratio in obese animals^11^. We observed the same tendency in this study irrespective of vendor. Also, we found a clear increase in Proteobacteria in mice on HFD and WD, although less dramatic in C57BL/6J. This may reflect differential abundance of Sutterella^10^. The presence of the Proteobacteria Sutterella has been shown to be increased in human T2D compared to non-diabetics^32^ and HFD feeding leads to increased presence of the Proteobacteria phyla^30,32,33^. In addition, we found some diet-specific effects at the individual species level. For example, the Firmicute Lactococcus was highly abundant in HFD but not in WD and chow, suggesting Lactococcus is not associated with obesity per se. Similarly, another Firmicute Allobaculum is mostly found in mice on WD. These differences may impact the differences in adiposity observed. However, in order to test this, additional experiments are needed, such as breeding all the C57BL/6J-related substrains in the same animal facility and micronutrient matched diets.

The C57BL/6J-related substrain differences observed in this study could be related to differential activity levels and energy expenditure which we did not measure in this study. Also, dietary composition, duration of diet intervention and the initial age of the animal may impact the response to DIO. For example, several reports have suggested that age impacts DIO and associated complications^24,34,35^. Accordingly, C57BL/6J mice were one week older compared to the other C57BL/6J-related substrains throughout the study, which may impact response to DIO. Also, it should be noted that the endpoint measurements of total body weight, tissue weight, liver TG, insulin levels, glucose levels, histology were performed with a two-hour offset, where C57BL/6BomTac was sacrificed first and C57BL/6J was sacrificed last. This may impact fasting insulin levels, liver TG levels and liver mass and may partly explain some substrain differences. For example, liver mass is reduced ~ 30% by prolonged fasting (24–72 h)^28^, possibly by loss of glycogen. Also, liver TG is increased by prolonged fasting^36^ and serum insulin levels are decreased^37^. However, these effects on fasting are primarily observed during nighttime fasting periods lasting more than 12 h in contrast to daytime fasting less than nine hours used in this study. Also, we did not find the same relative difference between C57BL/6J-related substrains in insulin levels and liver weight before diet intervention, suggesting that time of sacrifice is not confounding the differences observed between the C57BL/6J-related substrains.

Overall, these data show that C57BL/6J obtained a faster and more dramatic phenotype on WD compared to C57BL/6JBomTac and C57BL/6JRj when housed at identical environmental conditions. C57BL/6J stored excess fat more efficiently in adipose depots (both visceral and subcutaneous) and liver than C57BL/6JBomTac and C57BL/6JRj. Despite diet- and substrain-specific differences in body weight gain and storage of excess fat, and lower fasting insulin levels, there were no major differences in glucose and insulin tolerance between the three groups of mice after eight and nine weeks of intervention. Thus, the choice of C57BL/6J-related substrains in DIO studies mostly affects overall adiposity and TG storage in the liver with no clear correlation to the microbiota profile.

## Experimental procedures

### Experimental setup

Male mice six to seven weeks of age were sponsored by Taconic Bioscience (C57BL/6JBomTac, six weeks of age, animals were donated from a Taconic breeding facility in Denmark), Janvier Labs (C57BL/6JRj, six weeks of age, animals were donated from a breeding facility in France), and SCANBUR (C57BL/6J JAX stock #000664, distributed by SCANBUR and bred on Charles Rivers license, seven weeks of age, animals were donated from a breeding facility in Denmark). Mice were divided by weighted randomization (vendor blocked) into IVC-cages with three mice in each cage, acclimatized for two weeks, and maintained in 12-h light–dark cycle with lights on at 6 am (ZT0) and lights off at 6 pm (ZT12) with ad libitum access to chow (Altromin #1320 maintenance) and water. A total of 24 mice were donated by each vendor and acclimatized at the local mouse facility. Six mice were sacrificed for baseline measurements and 18 mice continued into the diet intervention experiment with 6 mice in each group. During the diet intervention experiment a few mice succumbed, which reduced the number mice in some of the groups. The number of mice used for the different measurements is indicated in the figure legends.

### Baseline group

A group of mice (n = 6) from each vendor was, following acclimatization, subjected to intraperitoneal glucose tolerance test (GTT), 3 days later DEXA scan (Lunar PIXImus, GE medical systems, USA) and the following day they were sacrificed.

### Diet intervention

After two weeks of acclimatization and sacrifice of the baseline group (week 0), a 10 week diet-intervention was initiated. The remaining mice were divided into three diet groups (n = 6) for C57BL/6J, C57BL/6JRj and C57BL/6JBomTac, respectively (nine groups in total), one group continued on a chow diet (Altromin #1324 maintenance, Brogaarden), a group switched to HFD (D12492, Research Diets) and one group switched to Western diet (#829100, Special diet service) supplemented with fructose (42 g/L of D-(-) Fructose, Sigma #F0127) in the drinking water. Nutrient composition of the diets is shown in Suppl. Table 1.

### Overview of tests during diet intervention

Body weight was measured every week of diet intervention. At week one and eight of diet intervention food and water intake were estimated in the following way: mice were put into clean cages, food was weighted, and volume drinking water measured. After three days, left-over food was weighted and remaining drinking water was measured. Based on this caloric intake was measured per cage and caloric intake for each mouse was estimated based on the number of mice in each cage. At week eight of diet intervention IP glucose tolerance test (GTT) was performed, at week two and nine DEXA scanning was performed and at week nine IP insulin tolerance test (ITT) was carried out. All mice on diet intervention were sacrificed after 10 weeks of diet intervention (Fig. 1b).

### Baseline and final sacrifice

At time of sacrifice body weight was recorded and the animals were euthanized by cervical dislocation. Trunk blood was collected for measurement of blood glucose levels. Selected tissues (i.e. pancreas, liver, epididymal white adipose tissue (eWAT, visceral depot), inguinal WAT (WAT, subcutaneous depot)), spleen, kidney, lumbar part of spine, and right hind leg) were dissected and weighted and either snap-frozen in liquid nitrogen or fixated in 4% PFA for 4 h-ON followed by 10–30% sucrose incubation for 4 h-ON followed by OCT-embedding.

At the baseline sacrifice C57BL/6JBomTac mice were sacrificed after 4 h (ZT6), C57BL/6JRj mice after 5,5 h (ZT7,5), and C57BL/6J mice after 7 h (ZT9) of fasting. At final sacrifice C57BL/6JBomTac were sacrificed after ~ 4 h (ZT6), C57BL/6JRj mice after 7 h (ZT9), and C57BL/6J after 9 h (ZT11) of fasting.

### GTT and ITT

Mice were fasted from ZT2 and GTT or ITT was performed for C57BL/6JBomTac at ZT4, C57BL/6JRj at ZT5,5 and C57BL/6J at ZT7. Body weights was measured and mice were subsequently injected intraperitoneally with either 20% D-glucose (2 g/kg) or insulin (Humulin R-100) (1 U/kg) according to standard operating procedures from MMPC concortium^38^. Blood glucose levels were measured using a glucometer (Freestyle Lite) after 15, 30, 60, and 120 min.

### Quantification of tissue mass by DEXA scan

The PIXImus 2 (GE Lunar) was used to obtain DEXA scans of live mice immobilized by isoflurane inhalation anesthesia and placed in the prone position with all four limbs stretched out. Version 2.10.041 of the proprietary software was used to acquire and analyze the scans. All body composition measurements were done on total body excluding the head and tail. DEXA images were also used to measure tibia length, as 1 pixel = 0.18 mm. Tibia length was estimated by converting tibia pixel from DEXA scan to length (1 pixel = 0.18 mm).

### Insulin and TG quantification

Insulin ELISA and TG quantifications were carried out essentially as described in^15,39^. In short, blood collected at baseline or final sacrifice were stored on ice and subsequently centrifuged at 10,000 rcf, 4 °C for 10 min. and prepared for insulin (Ultrasensitive Mouse Insulin ELISA kit, Chrystal Chem) or TG (Sigma, T2449) quantification according to manufacturer’s instructions. TG content calculated relative to tissue weight. HOMA-IR was calculated using HOMA calculator v2.2.3 (https://www.dtu.ox.ac.uk/homacalculator/).

### Liver glycogen measurements

10 mg liver tissue (n = 3–6) was homogenized in 100–500 ul water and boiled for 5 min. Insoluble materials were removed by centrifugation 5 min at 13,000×*g* 4 °C. Technical duplicates of 1 ul sample/well was prepared alongside a series of glycogen standard samples (0.4-2ug/well) following manufacturer’s instructions (Glycogen Assay Kit, Sigma-Aldrich, Cat#MAK016). Sample blanks omitting the glycogen hydrolysis step was included to remove the background signal from glucose. Data is presented as μg glycogen per mg liver tissue normalized to glycogen levels in chow fed mice from same vendor. Error bars represent standard deviation (SD) and statistical significance was calculated using Student’s t-test.

### Histology of liver and adipose tissue

Liver tissue was fixated in 4% paraformaldehyde for 4 h and subsequently in 20% sucrose ON at 4 °C. Oil Red O staining of liver tissue was performed on 5 µm cryosections.

Paraffin embedded adipose tissue was sectioned at 3 µm and stained with H&E on a DAKO CoverStainer (Agilent). The weighted mean volume of adipocytes was performed essentially as described in^40^. In short, adipocyte volume was determined from the H&E sections with Visiopharm Newcast stereology software; measurements on systematic, randomly selected fields were performed on at least 75 adipocyte profiles in each tissue and subsequently averaged giving one number per section.

### Feces and cecum sampling and DNA extraction

Fecal samples collected from mice throughout the project were stored at − 80 °C prior to the analysis. Feces were collected at week 4 and week 10 and samples from both timepoints were included in the analysis. Cecum samples were collected at the end of the study. The DNA from fecal content was extracted using Bead-Beat Micro AX Gravity Kit mod1 (A&A Biotechnology, Gdynia, Poland) according to the manufacturer’s instruction. DNA concentration and purity were measured using NanoDrop ND-1000 spectrophotometer (Saveen and Werner AB, Sweden). Extracted DNA was diluted to 20 ng/µL prior to the library preparation.

### 16S rRNA gene amplicon sequencing and data processing

Microbiota composition was determined using tag-encoded 16S rRNA gene (V3-V4-region) MiSeq-based (Illumina, San Diego, CA) high throughput sequencing. The V3 region (size of ~ 190 bp) was amplified using the following primers NXt_388_F: (5′-TCGTCGGCAGCGTCAGATGT GTATAAGAGACAGACWCCTACGGGWGGCAGCAG-3′) and NXt_518_R: (5′-GTCTCGTGGGCTCGGAGATGTGTATAAGAGACAGATTACCGCGGCTGCTGG-3′). The primers are compatible with Nextera Index Kit (Illumina, CA, USA). PCR reactions, in a total volume of 25 μL, were run on a SureCycler 8800 (Agilent Technologies, CA, USA. Reactions contain 12 μL of PCRBIO HiFi polymerase (PCR Biosystems Ltd, London, UK), 0.5 μL of each primer (10 μM), 5 μL of genomic DNA (20 ng/μL) and nuclease-free water. The following amplification protocol was used: Initial denaturation at 95 °C for 2 min, 33 cycles of denaturation at 95 °C for 15 s, annealing of primer at 55 °C for 15 s and elongation at 72 °C for 20 s. A second PCR round was applied to incorporate primers with adapters and indexes. PCR reactions contained the following reagents: 12 μL of PCRBIO HiFi polymerase (PCR Biosystems Ltd, London, UK), 2 μL corresponding P5 and P7 primer (Nextera Index Kit), 2 μL PCR product and nuclease-free water for a total volume of 25 μL. The amplification protocol was as follows: 95 °C for 1 min; 12 cycles of 95 °C for 10 s, 55 °C for 20 s and 72 °C for 20 s; and 72 °C for 5 min (SureCycler 8,800, Agilent Technologies, USA, CA). The amplified fragments with adapters were purified and normalized using custom made normalizing magnetic beads solution. Normalized pooled library was sequenced using Illumina NextSeq (Illumina, CA, USA).

### Sequencing data analysis

The raw dataset containing pair-ended reads with corresponding quality scores were merged and trimmed using fastq_mergepairs and fastq_filter scripts implemented in the USEARCH^41^ pipeline as described previously^42^ Purging the dataset from chimeric reads and constructing zero radius Operational Taxonomic Units (zOTU) was conducted using the UNOISE^43^. The Greengenes (13.8) 16S rRNA gene collection was used as a reference database^44^. Quantitative Insight Into Microbial Ecology 2 (QIIME2)^45^. open source software package (2019.4.0) was used for subsequent analysis steps. Alpha diversity measures expressed with an observed species (sequence similarity 97% OTUs) value were computed for rarefied OTU tables (5,000 reads/sample) using the alpha rarefaction workflow. Differences in alpha diversity were determined using a t-test-based approach employing the non-parametric (Monte Carlo) method (999 permutations) implemented in the compare alpha diversity workflow. PCoA plots were generated with the Jackknifed Beta Diversity workflow based on 10 distance metrics calculated using 10 subsampled OTU tables. The number of sequences taken for each jackknifed subset was set to 85% of the sequence number within the most indigent sample (~ 5,000). Community differences (beta-diversity) were revealed by weighted, unweighted and generalized UniFrac distances. Permutational Multivariate Analysis of Variance (PERMANOVA) was used to evaluate group differences based on weighted and unweighted UniFrac distance matrices. The differences in taxa abundance were between categories were estimated with a statistic framework: analysis of composition of microbes (ANCOM) based on raw OTU-table^46^.

### Statistical analysis

Data was analyzed using one- or two-way ANOVA (with one-way ANOVA between groups and two-way ANOVA on repeated measures) at a n of 4–6 (depending on vendor and diet group) and where appropriate Tukey comparison was used. Significance level was set at p = 0.05 or indicated directly in the figure. All data are shown as mean with standard deviation (SD).

### Animal experiments

*Ethics statement*. All mouse work and experimental protocols were approved by the Danish Animal Experiment Inspectorate (Approval #2014–15-0201-00437) and all methods were carried out in accordance to guidelines and regulations.

## Supplementary information

Supplementary information.

## Data Availability

The authors will provide data and protocols upon request to corresponding author. Due to limited tissue sample size, the authors will restrict sample availability to collaborations within the scope of the experimental aim of the project.
