# Supplementary material for: C57BL/6J substrain differences in response to high-fat diet intervention
Source: Sci Rep. 2020 Aug 20;10:14052. doi: 10.1038/s41598-020-70765-w (PMC7441320; doi:10.1038/s41598-020-70765-w)
Supplement: Supplementary file 1 — Supplementary information. [file 41598_2020_70765_MOESM1_ESM.pdf]

Supplemental material

C57BL/6J substrain differences in response to high-fat diet intervention

Majken Storm Siersbæk, Nicholas Ditzel, Eva Kildall Hejbøl, Stine Marie Præstholm, Lasse Kruse Markussen, Fabio Avolio, Lingzi Li, Lasse Lehtonen, Axel Kornerup Hansen, Henrik Daa Schrøder, Lukasz Krych, Susanne Mandrup, Louise Langhorn, Peter Bollen and Lars Grøntved

Contents:

Supplementary Figure 1 – page 2

Supplementary Figure 2 – page 3

Supplementary Figure 3 – page 4

Supplementary Figure 4 – page 5

Supplementary Figure 5 – page 6

Supplementary Figure 6 – page 7

Supplementary Figure 7 – page 8

Supplementary Figure 8 – page 9

Supplementary Figure 9 – page 10

Supplementary Table 1 – page 11

Supplementary figure legends – page 12

Siersbæk et al_Suppl. Fig. 1

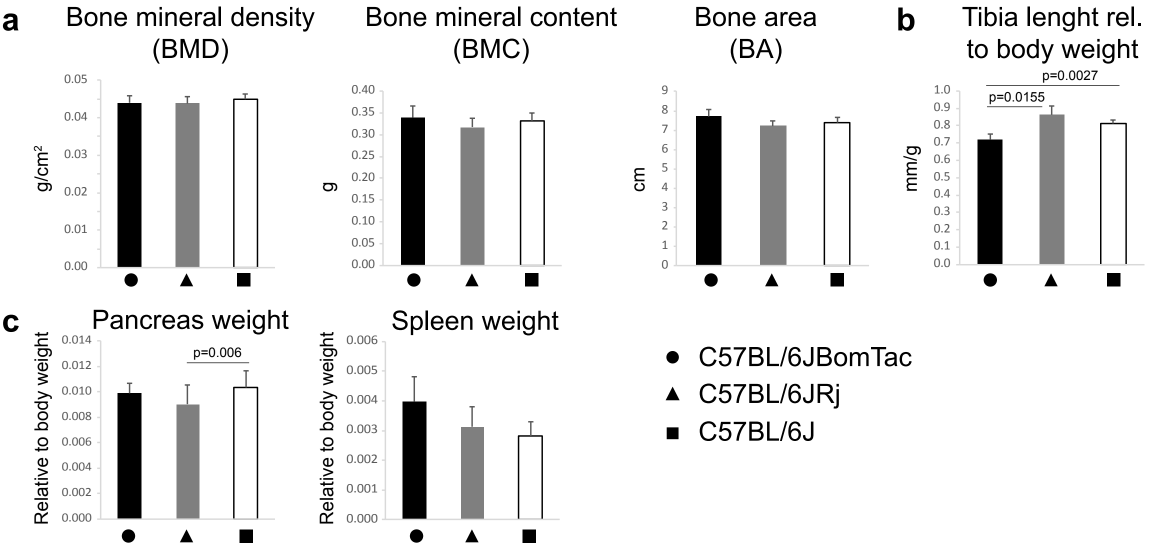

Siersbæk et al_Suppl. Fig. 2

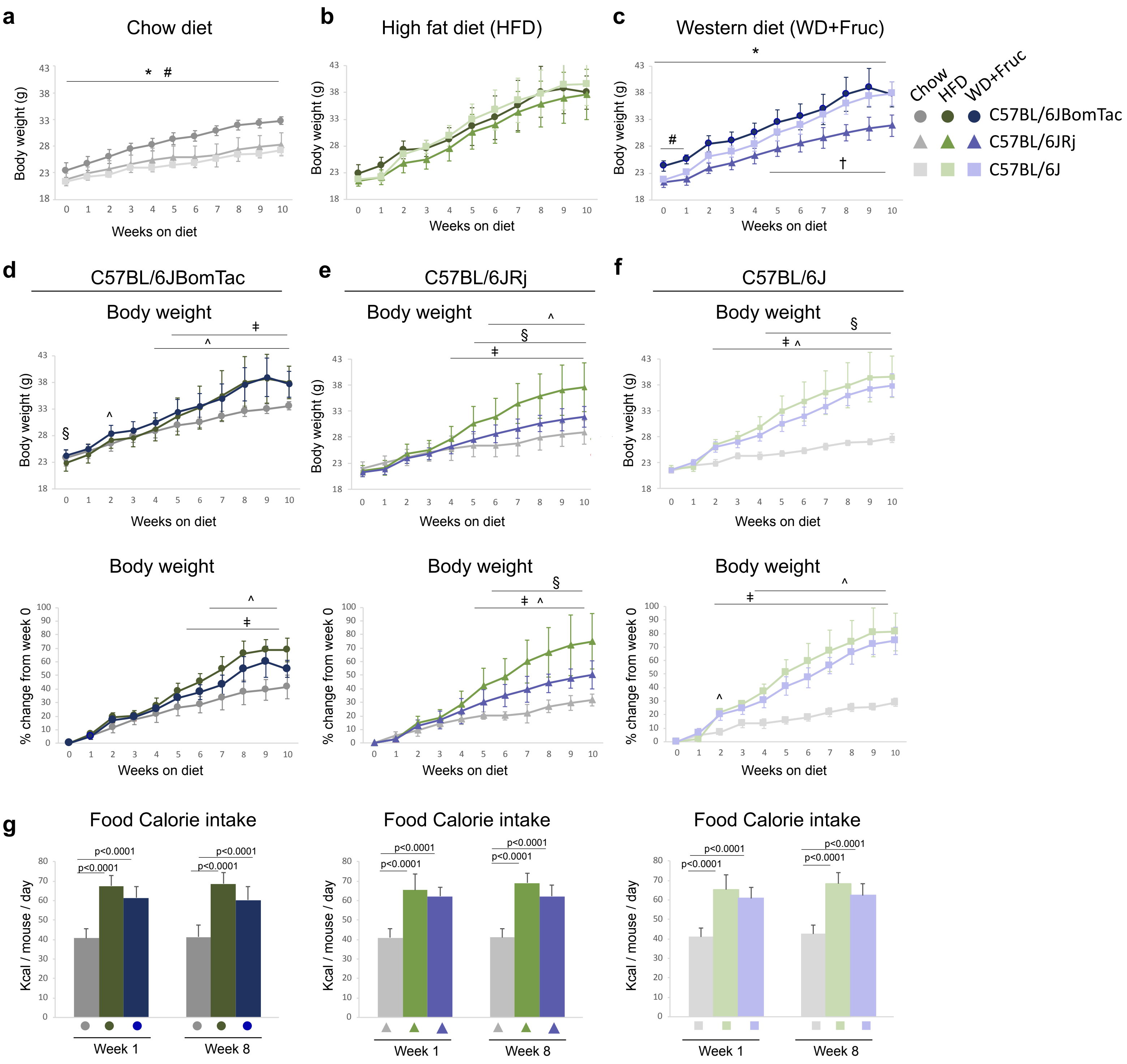

Siersbæk et al_Suppl. Fig. 3

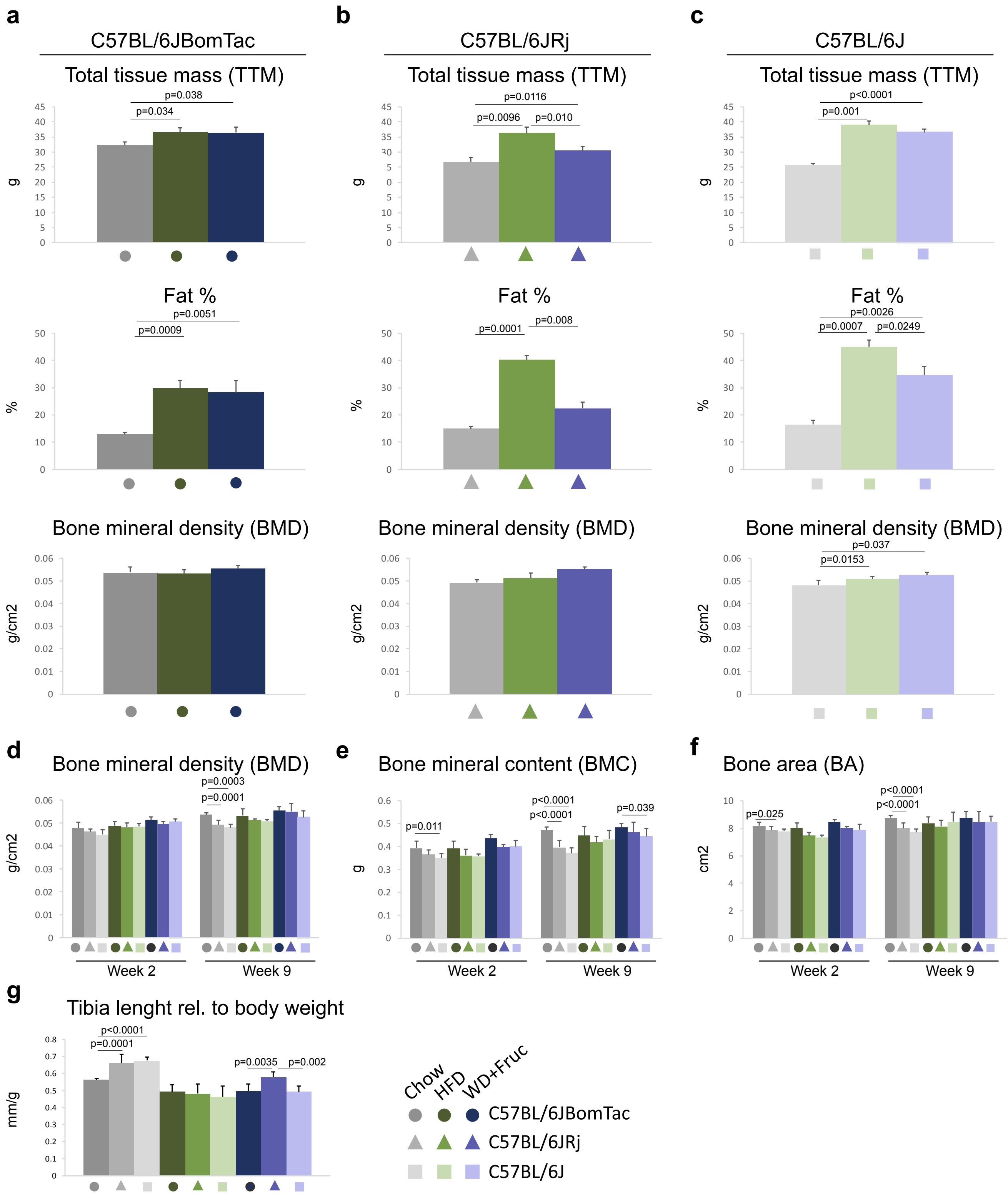

Siersbæk et al_Suppl. Fig. 4

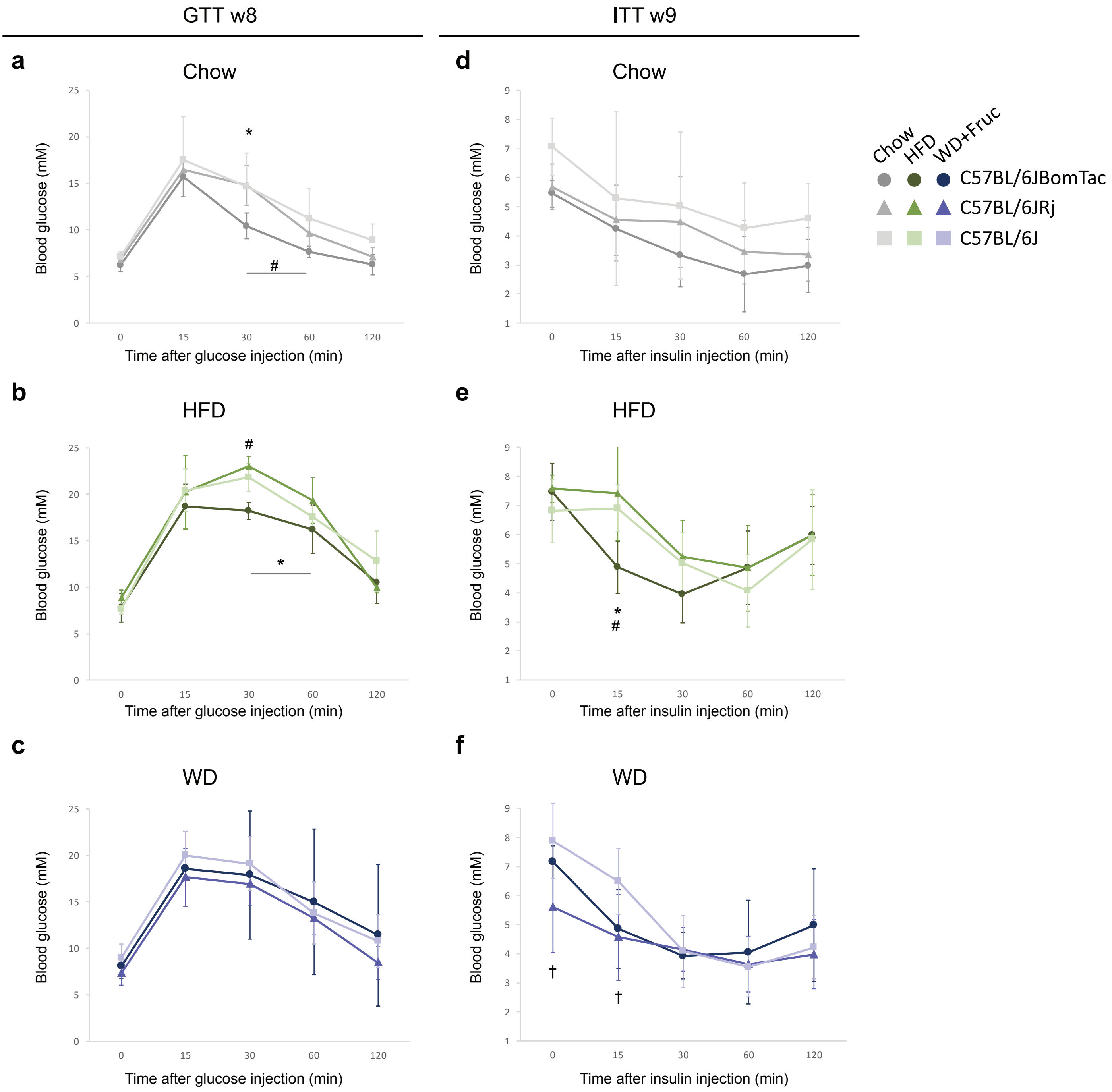

Siersbæk et al_Suppl. Fig. 5

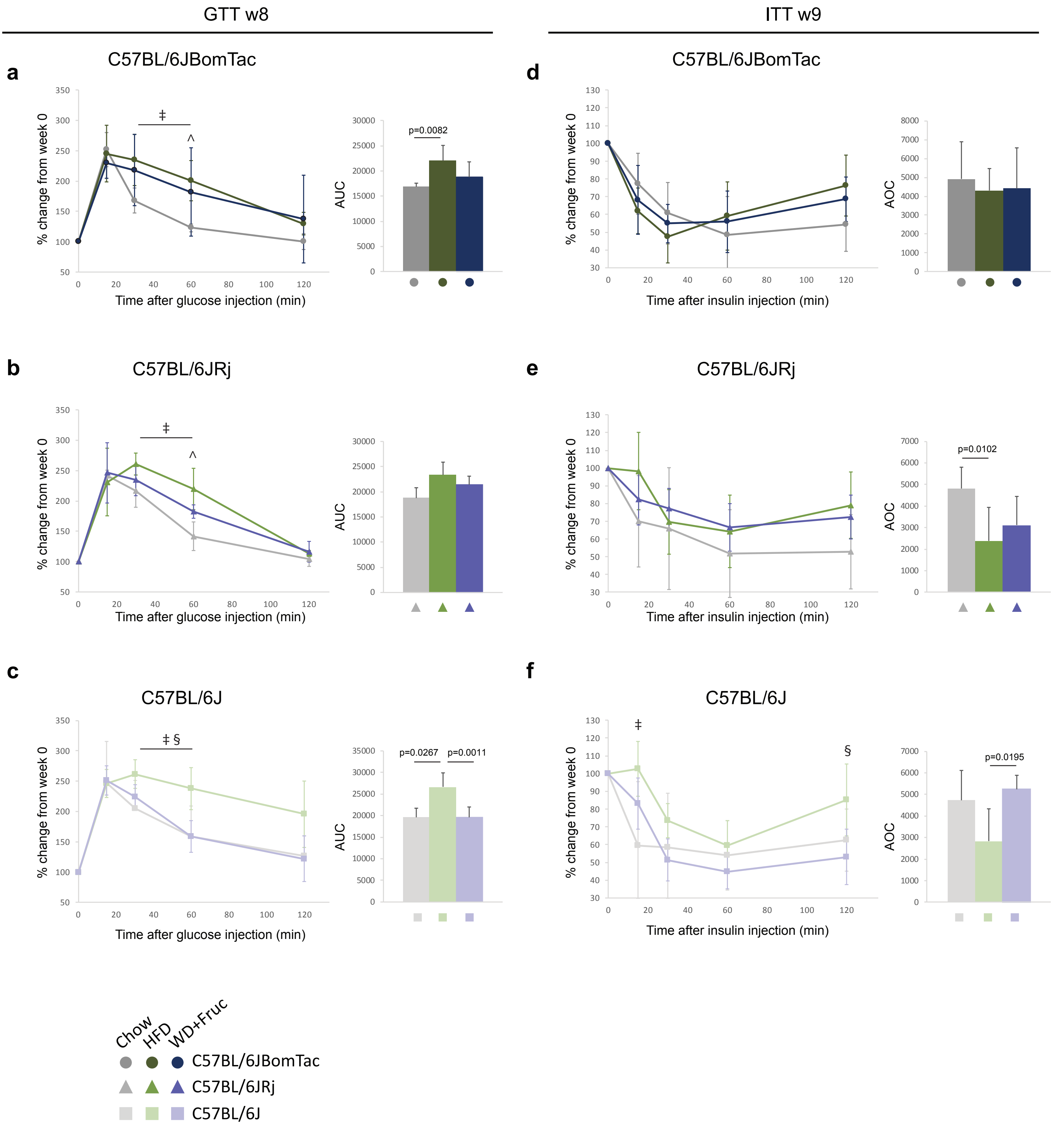

Siersbæk et al_Suppl. Fig. 6

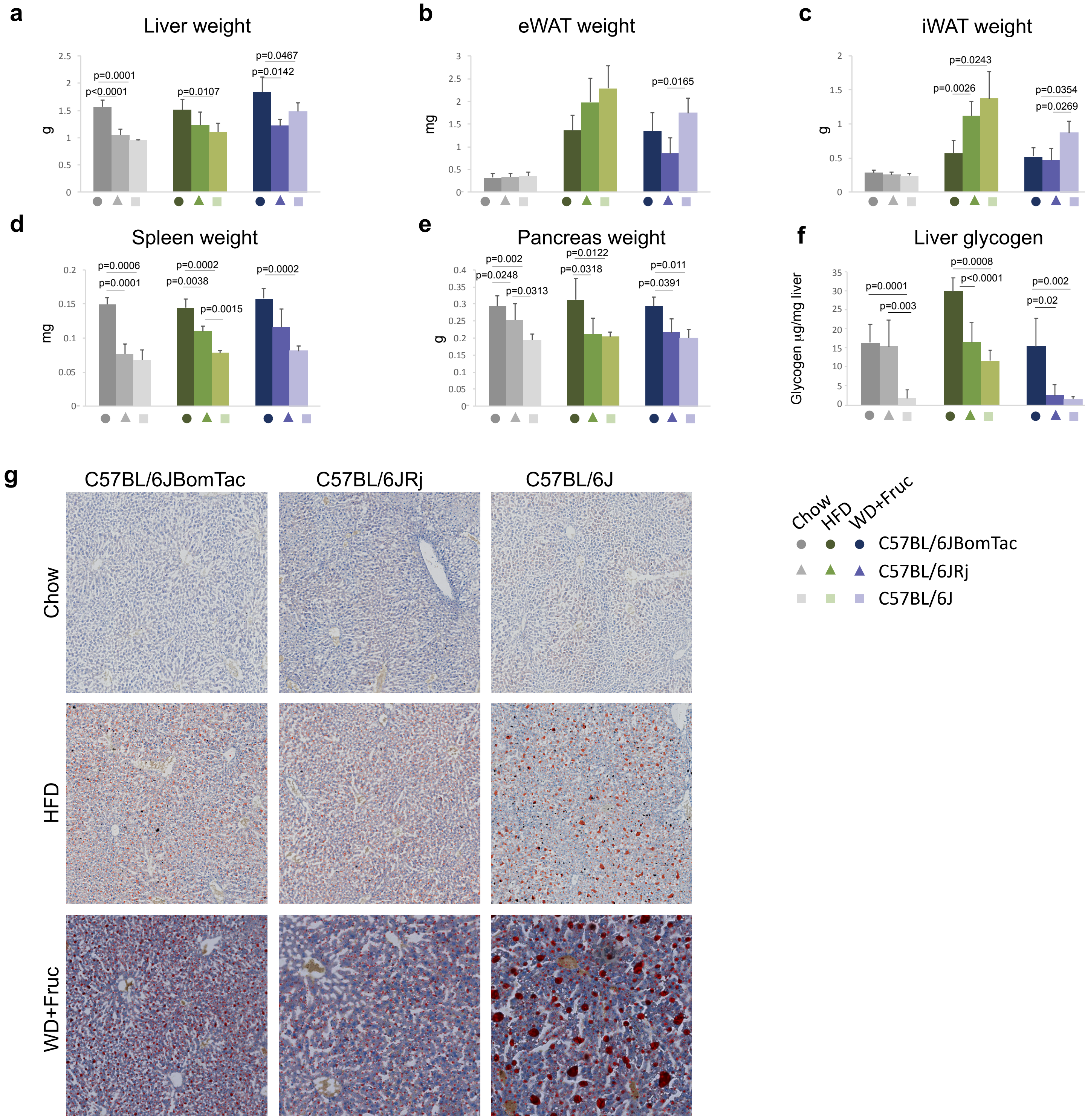

Siersbæk et al_Suppl. Fig. 7

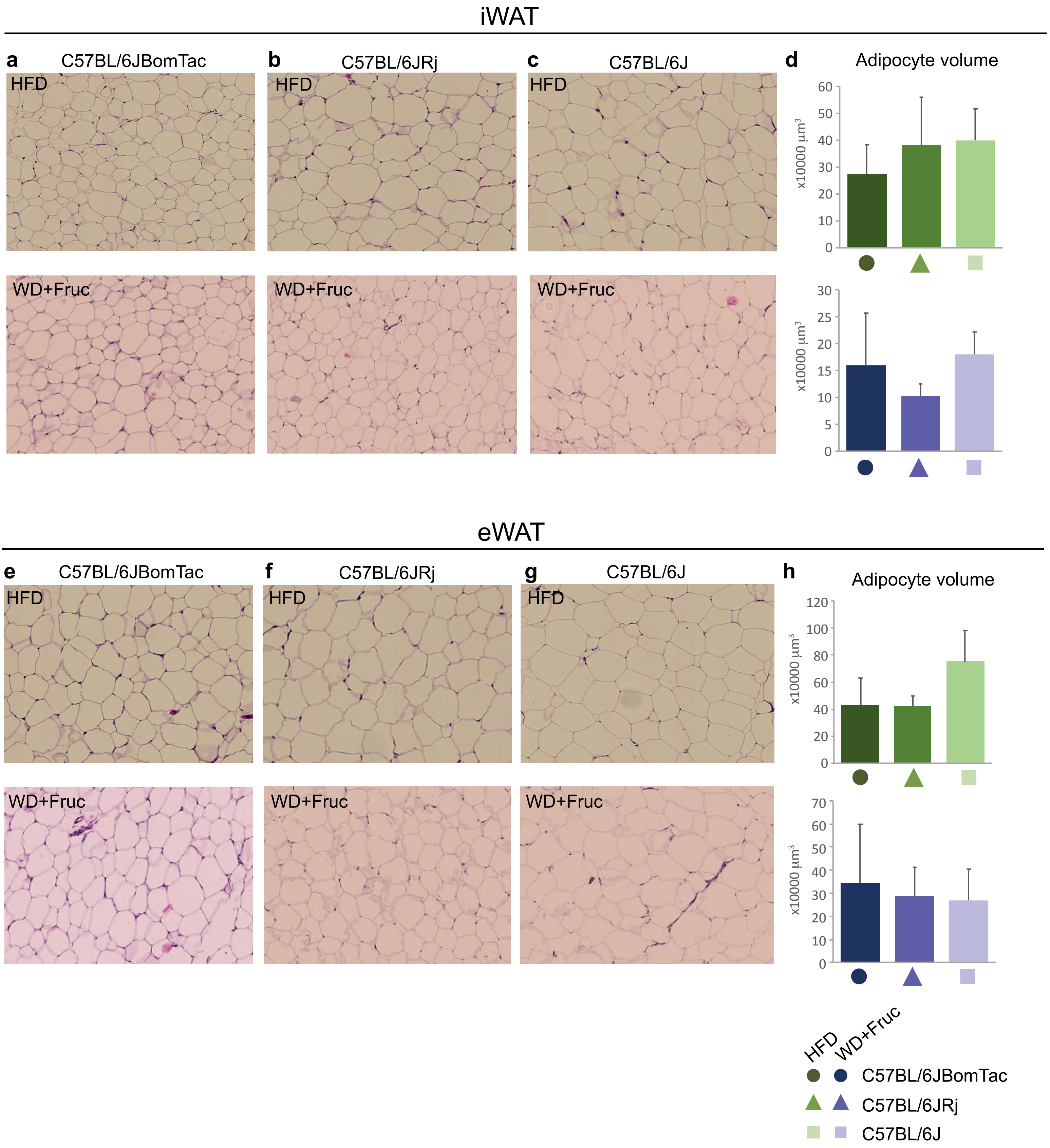

Siersbæk et al_Suppl. Fig. 8

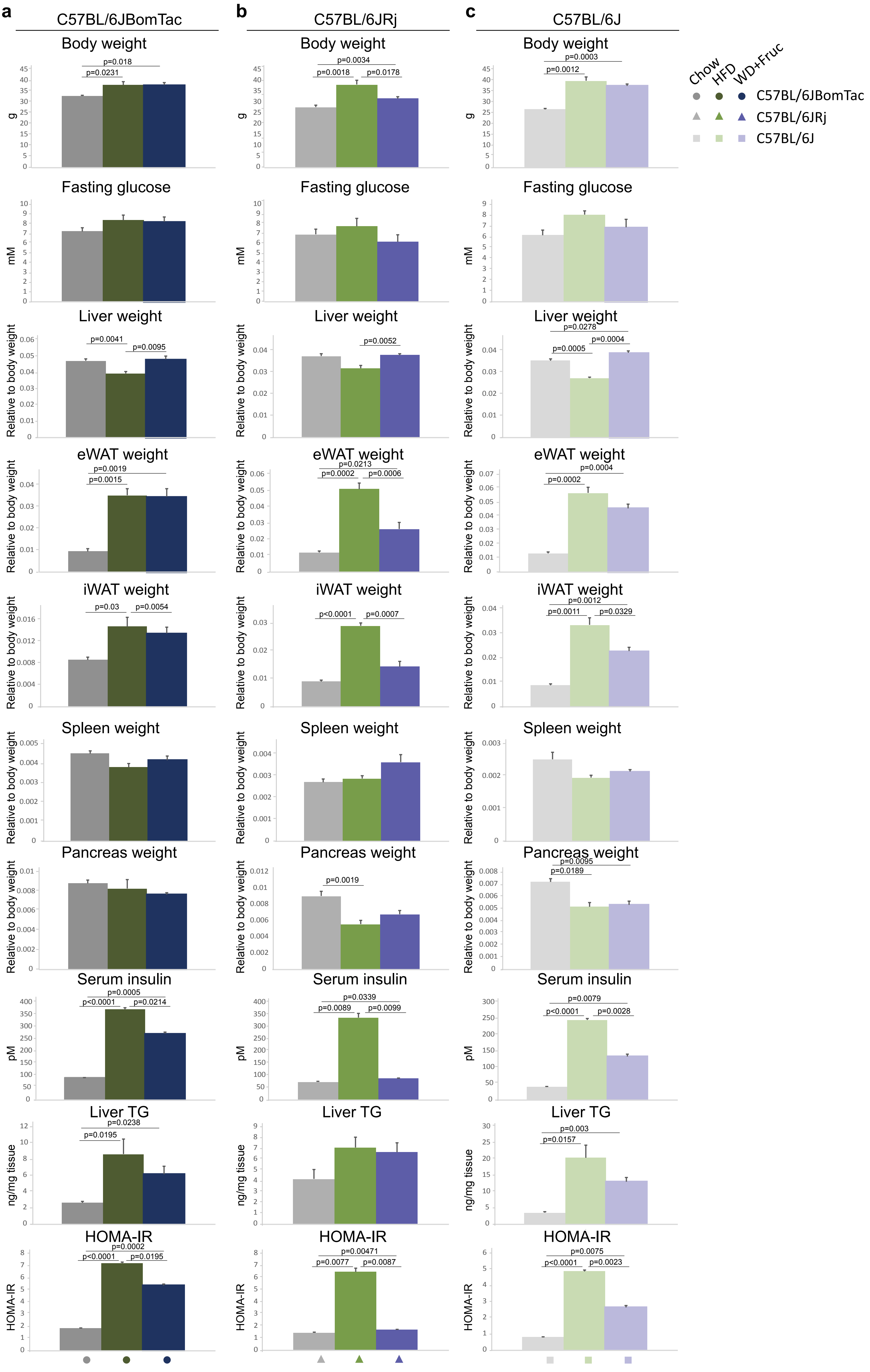

Siersbæk et al_Suppl. Fig. 9

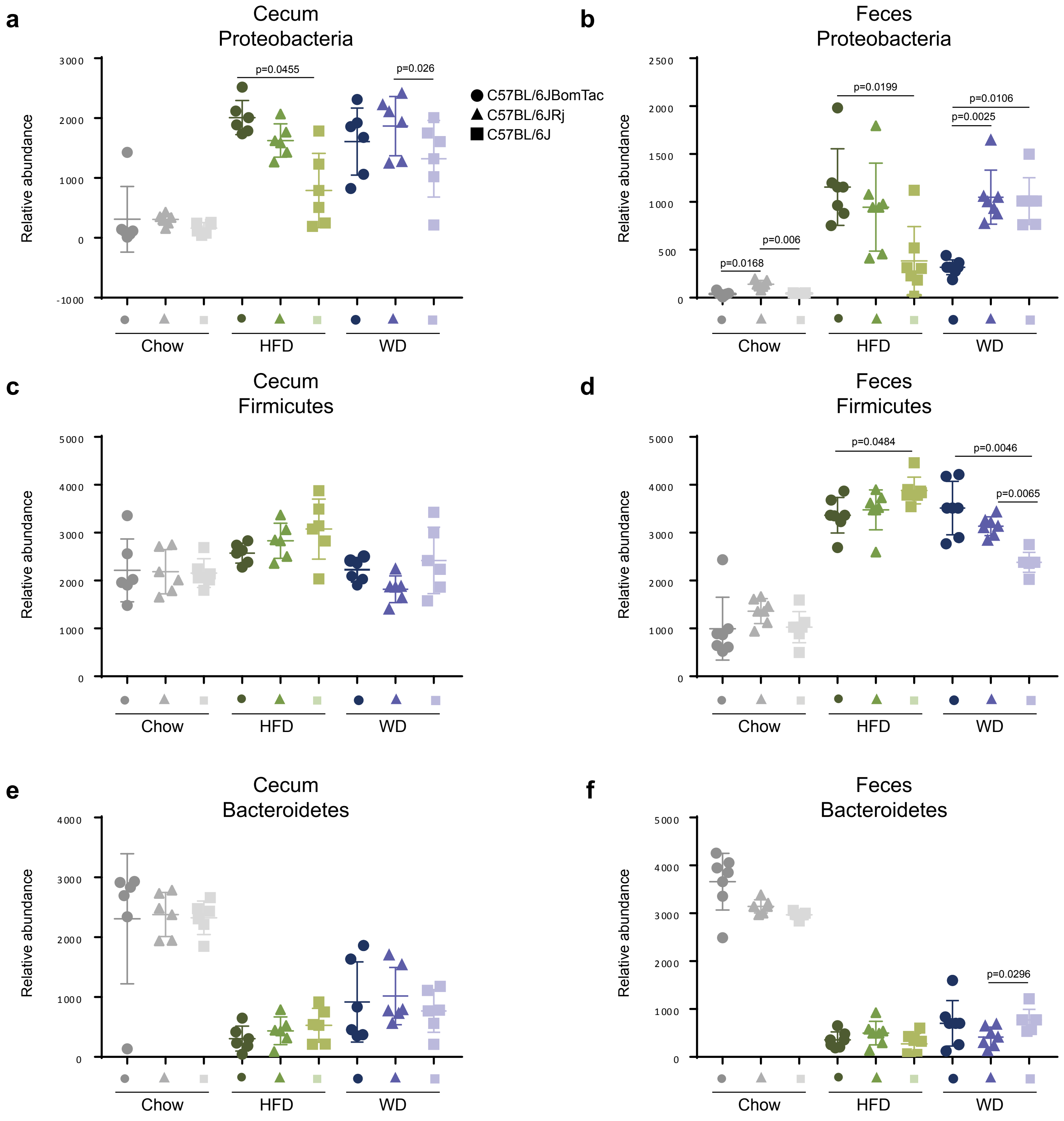

Siersbæk et al_Supplementary Table 1

a

	Chow diet			HFD			WD*		
	Altromin 1320 maintenance			D12492 (Research Diets)			Special diet service #829100		
Composi on		gm%	kcal%		gm%	kcal%		gm%	kcal%
Protein	Protein	19.2	24	Protein	26.2	20	Protein	17.5	15
Carbohydrate	Carbohydrate		64	Carbohydrate	26.3	20	Carbohydrate	50	43
Fat	Fat	4.1	12	Fat	34.9	60	Fat	21.4	42
Total	Total		100	Total		100	Total		100
kcal/gm	kcal/gm	3.27		kcal/gm	5.24		kcal/gm	4.83	

b

	Chow	HFD	WD*
**Fat source**	-	Lard (pork) and soybean oil	Milk fat and corn oil
**Crude nutrients (%)**			
Crude protein (N x 6.25)	19,2	24,4	17,5
Crude fat	4,1	34,6	21,4
Crude fiber	6,1	6	3,5
Crude ash	6,9	5,3	3,99
Starch	-	0,1	4,7
Carbohydrate	40,8	-	50
Sucrose	-	9,4	33,94
Dextrines	-	-	-
N free extracts	-	26,3	-
**Minerals (%)**			
Calcium	0,7	0,92	0,68
Phosphorus	0,5	0,64	0,46
Ca/P		1,44	-
Sodium	0,2	0,2	-
Magnesium	0,2	0,23	0,06
Potassium	0,9	0,97	0,36
**Fatty acids (%)**			
C 10:0		-	-
C 12:0		0,07	0,62
C 14:0		0,44	2
C 16:0		7,93	6,07
C 17:0		-	-
C 18:0		4,37	2,66
C 20:0		0,11	-
C 14:1		-	0,17
C 16:1		0,94	0,38
C 18:1		13,97	5,64
C 18:2		4,64	0,83
C 18:3		0,49	0,13
C 20:1		-	-
C 20:4		-	0,01
**Amino acids (%)**			
Lysine	0,8	2,02	1,07
Methionine	0,2	0,86	0,42
Cystein	0,3	0,45	0,34
Met+Cys	-	1,31	-
Threonine	0,6	1,07	0,59
Tryptophan	0,2	0,33	0,15
Arginine	1,2	0,95	0,44
Histidine	0,4	0,74	0,37
Valine	0,9	1,7	1,02
Isoleucine	0,8	1,38	0,86
Leucine	1,3	2,42	1,29
Phenylalanine	0,8	1,27	0,7
Phe+Tyr	-	2,56	-
Glycine	0,8	0,52	0,67
Glutamic acid	3,8	5,5	2,77
Aspartic acid	1,6	1,8	0,96
Proline	1,2	2,8	1,16
Serine	0,9	1,46	0,64
Alanine	0,9	0,81	0,54
**Vitamins (mg pr. kg)**			
Vitamin A (IU)	15000	15000	10050
Vitamin D3 (IU)	600	1500	1152
Vitamin E (IU)	75	150	62,39
Vitamin K	3	20	0,52
Vitamin C	36	-	0
Thiamine (B1)	18	25	5,75
Riboflavin (B2)	12	16	6,4
Pyridoxine (B6)	9	16	5,71
Cobalamin (B12) (ug)	24	30	10
Nicotinic acid	36	47	29,67
Pantothenic acid	21	55	15,81
Folic acid	2	16	1,91
Biotin (ug)	60	300	200
Choline	600	1140	822
**Trace elements (mg pr. kg)**			
Iron	198	168	47,75
Manganese	97	95	54,15
Zinc	94	65	53,08
Copper	13	13	7,5
Iodine	1,6	1,2	0,206
Selenium	0,3	0,2	0,16
Cobalt	0,4	-	0

* Mouse on WD also received 40g/L Fructose in the drinking water

Legends for supplementary figures

Suppl. Fig. 1

a) DEXA scan of C57BL/6JBomTac (●), C57BL/6JRj (▲), and C57BL/6J (■) before diet intervention quantifying bone mineral density (BMD) from bone mineral content (BMC) and bone are (BA). **b**) Tibia length relative to body weight. **c**) Weight of pancreas and spleen in baseline strain groups. Data is presented as mean ± SD. Statistical significance is calculated using one-way ANOVA with an n=6. P-values are shown in the figure.

Suppl. Fig. 2

a-c) Absolute body weight gain comparing chow, HFD, and WD+Fructose, respectively, C57BL/6JBomTac (●), C57BL/6JRj (▲), and C57BL/6J (■). In **d-f)** the absolute body weight gain (upper panel) and relative body weight gain, shown as percentage change from week 0 (lower panel), . **d**) C57BL/6JBomTac (●), **e**) C57BL/6JRj (▲), and **f**) C57BL/6J (■) mice on chow, HFD, and WD+Fructose. **g**) Estimated food calorie intake in C57BL/6JBomTac (●), C57BL/6JRj (▲), and C57BL/6J (■) mice on chow, HFD, and WD+Fructose. Data is presented as mean ± SD. Statistical significance is calculated using two-way ANOVA (body weight gain) or one-way ANOVA (calorie intake) with n=4-6. In a-c significance is indicated comparing the different strains p<0.05 ∗) C57BL/6JBomTac (●) vs. C57BL/6JRj (▲), #) C57BL/6JBomTac (●) vs. C57BL/6J (■) or †) C57BL/6JRj (▲) vs. C57BL/6J (■) or diets (ǂ) chow vs. HFD, (^) chow vs. WD or (§) HFD vs WD.

Suppl. Fig. 3

Total tissue mass (TTM), fat %, and bone mineral density (BMD) in **a**) C57BL/6JBomTac (●), **b**) C57BL/6JRj (▲), and **c**) C57BL/6J mice (■) at week nine of diet intervention. **d-g**) DEXA scan results showing **d**) bone mineral density (BMD), **e**) bone mineral content (BMC), and **f**) bone area in week two and nine of diet intervention and **g**) tibia length relative to body weight at week nine of diet intervention. Data is presented as mean ± SD. Statistical significance is calculated using one-way ANOVA with n=4-6. P-values are shown in the figure.

Suppl. Fig. 4

Glucose and insulin tolerance test, shown as absolute numbers of blood glucose concentration for individual C57BL/6J-related substrains fed chow (**a+d**), HFD **(b+e)** and WD **(c+f)** for C57BL/6JBomTac (●), C57BL/6JRj (▲), and C57BL/6J (■). Data is presented as mean ± SD. Statistical significance is calculated using two-way ANOVA with n=4-6. Significance is indicated comparing the different substrains at $p < 0.05$. ∗ and #) C57BL/6JBomTac (●) vs. C57BL/6J (■) or C57BL/6JRj (▲). †) C57BL/6J (■) vs. C57BL/6JRj (▲).

Suppl. Fig. 5

Glucose and insulin tolerance test, shown in percentage change from week 0 (left), and AUC/AOC (right) in **a+d**) C57BL/6JBomTac (●), **b+e**) C57BL/6JRj (▲), and **c+f)** C57BL/6J (■) mice on chow, HFD, and WD+fructose after eight and nine weeks of diet intervention. Data is presented as mean ± SD. Statistical significance for AUC/AOC is calculated using one-way ANOVA and two-way ANOVA for GTT/ITT time course with n=4-6. Significance is indicated comparing the different diets $p < 0,05$ (ǂ) $p < 0.05$ chow vs. HFD, (^) $p < 0.05$ chow vs. WD or (§) $p < 0.05$ HFD vs WD.

Suppl. Fig. 6

Absolute weight of **a**) liver, **b**) eWAT, **c**) iWAT, **d**) spleen, and **e**) pancreas in C57BL/6JBomTac (●), C57BL/6JRj (▲), and C57BL/6J (■) mice on chow, HFD, and WD+Fructose after 10 weeks of diet intervention. Data is presented as mean ± SD. Statistical significance is calculated using one-way ANOVA with n=4-6. P-values are shown in the figure. **f)** Liver glycogen level. Statistical significance is calculated using one-way ANOVA with n=4-6. P-values are shown in the figure. **g)** Representative Oil-Red-O staining of liver sections from one C57BL/6JBomTac, C57BL/6JRj, and C57BL/6J mouse on chow, HFD, or WD.

Suppl. Fig. 7

H&E staining of iWAT (**a-c**) and eWAT (**e-g**) from C57BL/6JBomTac (**a+e**), C57BL/6JRj (**b+f**), and C57BL/6J (**c+g**) mice. Pictures are representative of sections from three different mice. **d+h**) The weighted mean volume of adipocytes in iWAT and eWAT determined from H&E sections by measuring and averaging at least 75 adipocytes of each tissue.

Suppl. Fig. 8

After 10 weeks of diet intervention the body weight, fasting glucose, weight of liver, eWAT, iWAT, spleen, pancreas, fasting serum insulin, and liver TG levels were measured, and HOMA-IR calculated in **a**) C57BL/6JBomTac (●), **b**) C57BL/6JRj (▲), and **c**) C57BL/6J mice (■) on chow, HFD, and WD+Fructose. Data is presented as mean ± SD. Statistical significance is calculated using one-way ANOVA with n=4-6. P-values are shown in the figure.

Suppl. Fig. 9

Relative abundance of specific microbiota within C57BL/6JBomTac (●), C57BL/6JRj (▲), and C57BL/6J (■) groups on chow, HFD, and WD+Fructose. Proteobacteria (**a+b**), Firmicutes (**c+d**), and Bacteroidetes (**e+f**) are shown for cecum and feces samples. Data is presented as mean ± SD. Statistical significance is calculated using one-way ANOVA with n=5-6. P-values are shown in the figure.

Suppl. Table 1

Nutrient composition of chow, HFD and WD. **a)** Macronutrients in gm% (weight percentage) and kcal% (caloric percentage). **b)** Micronutrients in weight percentage or mg per kg.
